# A General-Purpose Machine Learning R Library for Sparse Kernels Methods With an Application for Genome-Based Prediction

**DOI:** 10.3389/fgene.2022.887643

**Published:** 2022-06-03

**Authors:** Osval Antonio Montesinos López, Brandon Alejandro Mosqueda González, Abel Palafox González, Abelardo Montesinos López, José Crossa

**Affiliations:** ^1^ Facultad de Telemática, Universidad de Colima, Colima, Mexico; ^2^ Centro de Investigación en Computación (CIC), Instituto Politécnico Nacional (IPN), México City, Mexico; ^3^ Centro Universitario de Ciencias Exactas e Ingenierías (CUCEI), Universidad de Guadalajara, Guadalajara, Mexico; ^4^ International Maize and Wheat Improvement Center (CIMMYT), Texcoco, Mexico; ^5^ Colegio de Postgraduados, Montecillo, Mexico

**Keywords:** r package, machine learning, kernel, supervised learning, sparse kernels, genome-base prediction

## Abstract

The adoption of machine learning frameworks in areas beyond computer science have been facilitated by the development of user-friendly software tools that do not require an advanced understanding of computer programming. In this paper, we present a new package (sparse kernel methods, SKM) software developed in R language for implementing six (generalized boosted machines, generalized linear models, support vector machines, random forest, Bayesian regression models and deep neural networks) of the most popular supervised machine learning algorithms with the optional use of sparse kernels. The SKM focuses on user simplicity, as it does not try to include all the available machine learning algorithms, but rather the most important aspects of these six algorithms in an easy-to-understand format. Another relevant contribution of this package is a function for the computation of seven different kernels. These are Linear, Polynomial, Sigmoid, Gaussian, Exponential, Arc-Cosine 1 and Arc-Cosine L (with L = 2, 3, … ) and their sparse versions, which allow users to create kernel machines without modifying the statistical machine learning algorithm. It is important to point out that the main contribution of our package resides in the functionality for the computation of the sparse version of seven basic kernels, which is indispensable for reducing computational resources to implement kernel machine learning methods without a significant loss in prediction performance. Performance of the SKM is evaluated in a genome-based prediction framework using both a maize and wheat data set. As such, the use of this package is not restricted to genome prediction problems, and can be used in many different applications.

## Introduction

Machine learning has become the main approach for solving complex, data-based problems and it is being used everywhere from devices and digital services such as smartphones and websites, to scientific research in various fields (Wang et al., 2016; Ott et al., 2020; Shahin et al., 2020; Montesinos-López et al., 2021a). As machine learning research has progressed, so has the supply and demand of software that facilitates its implementation. For this reason, numerous open-source packages for data related tasks and machine learning algorithms have become even more prevalent (Abadi et al., 2015; Wickham et al., 2015; Pandas development team, 2020).

One of the most used programming languages for data analysis is R (R Core Team, 2021) due to its statistical computing focus, free and open-source software and the thousands of packages that extend its power to all kind of analysis and related tasks of data science. In fact, it is difficult to find a machine learning algorithm not implemented within an R package. Likewise, it can even be said that some of the R packages contain more complete/specialized implementations (Ishwaran et al., 2008; Friedman et al., 2010; Meyer et al., 2019) than those available in other programming languages. As machine learning is strongly based on statistical models and R is the *de facto* language for statistics research, those who embark on machine learning will encounter R at some point.

Most R packages of machine learning algorithms include one type of model or a family of similar models. While using R packages have clear advantages, there are some challenges. For example, each package has been developed by different authors and there is no standardized code style guideline. This complicates the use of packages since it requires users to learn the expected data format, the name and expected parameters and the code convention (if any) in order to train a model or retrieve outputs. In addition, several complementing packages may be needed to perform cross validation of models, hyperparameter tuning, and compute accuracy metrics, among others. There are some libraries that seek to integrate a wide range of tools needed for machine learning in one place, such as scikit-learn (Pedregosa et al., 2011) in Python; H2O in Java (with both R and Python versions); and caret (Kuhn, 2016), mlr3 (Lang et al., 2019) and tidy models (Kuhn and Wickham, 2020) in R. All these options have their own philosophy, and they were designed using diverse approaches to implement machine learning models.

We consider the mlr3 as the most powerful R package for machine learning because of its potential scope. The mlr3 package is an object-oriented solution for machine learning focused on extensibility since it does not implement any model itself, but rather provide a unified interface for many existing packages in R. While this is a major advantage, such an approach does not completely solve the dependency of other packages, which require knowledge of both the package that implements the model and mlr3. It is worthwhile to learn how to use all the components in the mlr3 environment because it also provides efficient implementation of most data related tasks, parallelization, hyperparameter tuning and feature selection, among others. However, it takes times getting accustomed to the way mlr3 works and how things are defined in parts with the object-oriented paradigm, which is not so common in R programming. Nevertheless, this learning curve is relatively short.

Alternatively, we have caret and tidy models providing their own standardized interface, which is a very important factor in a good quality software. Like mlr3, these two packages use other third party packages of machine learning algorithms in tandem to train models as they provide with different options for the same algorithm. Caret is the oldest of these three packages, and as such, it still enjoys considerable popularity. Notwithstanding, the major advantage of tidy models is that they belong to the tidy verse, a collection of R packages tailored for data science that share an underlying design philosophy, grammar and data structures (Wickham et al., 2019); consequently, if users are familiar with tidy verse packages, they will naturally start using tidy models.

In the current paper, we present SKM (Sparse Kernels Methods), a new R package for machine learning that includes functions for model training, tuning, prediction, metrics evaluation and sparse kernels computation. The main goal of this package is to provide a stand-alone (or self-contained) R software, focused on the austere implementation of only six basic supervised learning models that are easy to understand from the user´s point-of- view. We will focus specifically on six types of supervised models, which are explained in the next section. The model functions in SKM were designed with simplicity in mind, and as such, the parameters, hyperparameters and tuning specifications are defined directly when calling the function; subsequently, users can understand how the package works by observing a handful of examples. Furthermore, we strive to provide clear documentation following a base convention in the functions. Likewise, all the parameters are validated with checkmate software (Lang, 2017) to inform the user when an error occurs through meaningful error messages—something that many other packages neglect. The most important hyperparameters of each model can be tuned with two different methods: grid search and Bayesian optimization (Osborne et al., 2009) based on the code of Bayesian Optimization package (Yan, 2016). Although Bayesian optimization is a very popular and effective method of tuning, the mlr3 and caret packages do not offer this option.

Kernels have proven to be useful in helping the conventional machine learning algorithms capture non-linear patterns in data (Montesinos-López et al., 2021b; Montesinos-López et al., 2022a). In addition to capturing complex non-linear patterns, the sparse kernel version of kernel methods can also save significant computational resources without a relevant loss in prediction accuracy (Montesinos-López et al., 2021b; Montesinos-López, et al., 2022a). In this paper by sparse kernels we define those kernels that are built with only a fraction of the total amount of inputs by assuming that the input matrix is a sparse matrix, that is, a matrix that contain many information with zeros. For this reason, the term level of compression, here is used, as one minus the proportion of the total lines (or rows) used to compute the sparse kernels thus representing the level of dimensionality reduction reached by using these sparse kernels. To the best of our knowledge, there is no existing R package for the computation of dense kernels and sparse kernels (that compress the dimension of the dense kernels), which is the added value of SKM and what gives it its name. The approach of sparse kernels implemented in the SKM library is based on the method proposed in Cuevas et al. (2020).

As software developers and consumers, we are aware of the importance of sharing our work with the community, and as such, SKM is a completely open-source software released under the GNU Lesser General Public License v3.0 (LGPLv3). As such, anyone can explore the source code, make modifications and build on it to develop other tools.

## Machine Learning Algorithms

The SKM package includes six different functions of supervised machine learning algorithms. Table 1 shows the six models that can be implemented under the SKM package, and the package of origin that each of these models uses, in addition to the function to implement these models in the SKM library.

**TABLE 1:** Models that can be implemented in the SKM library.

Model	Name	Package of origin	Function in SKM	Response variables
M1	Generalized boosted machines	gbm (Greenwell et al., 2020)	generalized_	Binary, categorical and continuous; only univariate
			boosted_machine ()	
M2	Generalized linear models	Glmnet (Friedman et al., 2010)	Generalized	Binary, categorical, continuous, and count; univariate and multivariate only for continuous response variables
			_linear_model ( )	
M3	Support vector machines	e1071 (Meyer, et al., 2019)	Support	Binary, categorical and continuous, only for univariate response variables
			_vector_machine ()	
M4	Random forest	RandomForestSRC (Ishwaran, et al., 2008)	random_forest ()	Binary, categorical and continuous, univariate and multivariate
M5	Bayesian regression models	BGLR (Perez and de los Campos, 2014)	bayesian_model ()	Binary, categorical and continuous, univariate and multivariate only for continuous response variables
M6	Deep neural networks	keras (Allaire and Chollet, 2016)	deep_learning ()	Binary, categorical, continuous, and count; univariate and multivariate for all response variables

It is important to point out that all models that can be implemented in the SKM library will be able to implement the seven kernels methods and its sparse versions explained in the next section, whereas in the case of deep neural networks (M6), only fully connected networks can be implemented. Under the Bayesian methods, Bayesian Ridge regression (BRR), Bayes A (Bayes_A), Bayes B (Bayes_B), Bayes C (Bayes_C), Bayesian Lasso (Bayes_Lasso) and the best linear unbiased predictor (GBLUP) in its Bayesian version (BGBLUP) can be implanted. It should be highlighted that the six models that can be implemented in the SKM library, including all the Bayesian methods available in model M5, can also work with kernels. First, the matrix of inputs (X) is created; then the square root of the kernel is computed; next the design matrix of lines is post-multiplied by the square root of the kernel; and finally this design matrix is used as input in any of the six models when kernels are used. The exception is under the BGBLUP in model M5, where the computed kernels are directly used.

The additional layer of abstraction allows all functions to share the same data input format. Internally, data is adapted to the expected format of each package, where the result and prediction objects returned by these functions are also in the same format. Another benefit of these functions is that some parameters that can be inferred from data itself do not need to be supplied by the user, rather they are set automatically. For example, the family parameter of glmnet package which has to be “Gaussian” for continuous response variables, “binomial” for binary variables, “multinomial” for categorical response variables and “Poisson” for count variables, can be inferred from the response variable. In addition, the same functions permit hyperparameter tuning in an easy and user-friendly format without the need to call another function or initiate another object. In theory, as with all packages that internally call functions of other packages, ease of use and extended functionality is expected to improve with a slight increase in computational demand for the extra operations required. Furthermore, since these operations are of computationally low cost, there is no significant loss of power.

Supplementary Appendix SA included some comparative examples of the equivalent implementation of some machine learning models with mlr3, SKM and randomForestSRC, the original package.

## Sparse Kernels

As Montesinos-López et al. (2021b) point out, kernel methods transform the independent variables (inputs) using a kernel function, followed by the application of conventional machine learning techniques to the transformed data to achieve better results, mainly when the inputs contain non-linear patterns. Kernel methods are excellent options in terms of computational efficiency when managing large, complex data that show non-linear patterns; likewise they can be used with any type of predictive machine. Consequently, we have included the kernelize function in SKM that can compute the same 7 kernels and their sparse versions as described in Montesinos-López et al. (2021b): Linear, Polynomial, Sigmoid, Gaussian, Exponential, Arc-Cosine 1 and Arc-Cosine L (with L = 2, 3, … ). The kernel computation is independent from the model fitting process, which allows the kernelize function to be used with other packages or conversely, the machine learning algorithms implementation of SKM can be used without kernels.

Next the algorithm to approximate the kernels, here called sparse kernels is described in general terms. We assume that the response variable (y) is associated to the genomic effects (u) as:

y=μ1+u+e (1)

where μ is the overall mean, **1** is the vector of ones, and y is the vector of size n . Moreover, u is the vector of genomic effects $u∼N\left(0,\sigma_{u}^{2}K\right)$ , where $\sigma_{u}^{2}$ is the genomic variance component and matrix K is the dense kernel of order n×n constructed with any of the kernel methods explained above. The random residuals are assumed independent with normal distribution $e∼N\left(0,\sigma_{e}^{2}I\right)$ , where $\sigma_{e}^{2}$ is the error variance. The dense kernel, K , can be approximated as $K\approx Q=K_{n,m}K_{m,m}^{-1}K_{n,m}^{\prime }\;\;\;$ (Williams and Seeger, 2001), where Q will have the rank of $K_{m,m}$ , that is, m . The computation of this kernel is facilitated since it is not necessary to compute and store the original matrix K , since only $K_{m,m}$ and $K_{n,m}$ are required. This approximation of the dense kernel (which we call sparse kernel) use m out of n lines to compute $K_{m,m}^{-1}$ , then an eigen-value-decomposition of $\;K_{m,m}^{-1}=US^{-1/2}S^{-1/2}U^{\prime }$ is used, where U are the eigen vectors of order m×m and $S_{m,m}$ is a diagonal matrix of order m×m with the eigen values ordered from largest to smallest. Next, these values are substituted in $Q=K_{n,m}US^{-1/2}S^{-1/2}U^{\prime }K_{n,m}^{\prime }$ resulting in $u∼N\left(0,\sigma_{u}^{2}K_{n,m}US^{-1/2}S^{-1/2}U^{\prime }K_{n,m}^{\prime }\;\;\;\;\right)$ , and thus, model (1) can be expressed as:

$$y=µ1_{n}+Pf+\varepsilon$$ (2)

Model (2) is similar to model (1), except that f is a vector of order m×1 with a normal distribution of the form $f∼N\left(0,\sigma_{f}^{2}I_{m,m}\right)$ , where $P=K_{m,n}US^{-1/2}$ is now the design matrix. This implies estimating only m effects that are projected into the n dimensional space in order to predict u and explain y . Note that model (2) can be implemented under a conventional mixed model framework or under any statistical machine learning algorithm assuming that the f term of Equation 2 is a fixed effect. For example, under a linear kernel the $K_{m,n}$ and $K_{m,m}$ can be computed as $K_{m,m}=\frac{X_{m,p}X_{m,p}^{\prime }}{p}\;\;$ and $K_{n,m}=\frac{X_{n,p}X_{m,p}^{\prime }}{p}\;\;\;$ respectively, where $X_{m,p}$ is the centered and scaled matrix of markers with m lines and p markers, and $X_{n,p}$ is the centered and scaled matrix of markers with n lines and p markers. In summary, according to Cuevas et al. (2020), the approximation described above consists of the following steps:

Step 1: Computing the following matrices, matrix $K_{m,m}$ from m lines of the training set.
Step 2: Constructing matrix $K_{n,m}$

Step 3: Eigen value decomposition of $K_{m,m}$

Step 4: Computing matrix $P=K_{n,m}US^{-1/2}$ .
Step 5: Fitting the model under any of the above mentioned statistical machine learning using $P=K_{n,m}US^{-1/2}$ as design matrix and y as response variable.

One of the major advantages of the sparse kernels is data dimensionality reduction since the number of parameters to be estimated is reduced significantly in comparison to the dense kernels. This is useful when working with high dimensional data where the number of columns is considerably greater than the number of rows, as there is few data, and the training process of the model is more efficient. More details about the kernels and the approximated kernels, here called sparse kernels, that were implemented in the SKM library can be found in detail in Montesinos-López et al. (2021b) and Montesinos-López, et al. (2022a).

In Supplementary Appendix SB we have included some examples of how to use the kernelize function of SKM to compute the different kernels and their sparse versions.

## Evaluation Metrics

Evaluating models’ performance is an important task of all machine learning workflows. For this reason, in SKM we have included functions of the most popular metrics to evaluate models’ performance for both regression and classification problems. The regression metrics included are: Mean Squared Error (MSE), Root Mean Squared Error (RMSE), Normalized Root Mean Squared Error (NRMSE, with four types of normalization: by standard deviation, mean, range and interquartile range), Mean Absolute Error (MAE) and Mean Arctangent Absolute Percentage Error (MAAPE). The classification metrics included are: accuracy, specificity, sensitivity, Kappa coefficient, Brier score, Matthews correlation coefficient, precision, recall, Area Under the ROC Curve (ROC-AUC), Precision-Recall Area Under the Curve (PR-AUC), F1 score and a function to compute the confusion matrix. In addition to the functions already mentioned, the wrapper functions numeric summary and categorical summary compute all the regression and classification metrics to obtain a complete summary of the model’s performance in a simple function. More details about most of these metrics can be found in chapter 4 (Overfitting, model tuning and evaluation of prediction performance) of the book Multivariate statistical machine learning methods for genomic prediction (Montesinos-López et al., 2022b).

As expected, all these metric functions work in harmony with the machine learning algorithm functions since they use the same data format; no extra data processing is necessary when they are used correctly. This does not limit or complicate their use with other packages, as shown in the detailed documentation provided.

Supplementary Appendices SA, SB include examples of some metric functions that receive the observed and predicted values (or probabilities in classification) and return a numeric value.

## Installation

SKM is a package built for the R ecosystem. As an open source project, the package has first been published in a GitHub repository at https://github.com/brandon-mosqueda/SKM where the full source code and another option of installing the development version (and most updated) can be found. This development version may include corrections of reported bugs and new functionalities, among others. Likewise, in the repository users can also find a place to report bugs or contribute to the project. In order to install the development version, the following commands must be executed in an R terminal.

devtools::install_github ("cran/randomForestSRC")

devtools::install_github ("gdlc/BGLR-R")

devtools::install_github ("rstudio/tensorflow")

if (!require ("devtools")) {install.packages ("devtools")}

devtools::install_github ("brandon-mosqueda/SKM")

## Illustrative Examples

Next, we will illustrate the use of the SKM library with two popular data sets in genomic selection using 5-random partitions to evaluate the prediction performance with the two available tuning options. The response variables in both datasets are numeric response variables, and as such, we present the prediction performance in terms of Mean Arctangent Absolute Percentage Error (MAAPE), Mean Absolute Error (MAE), Mean Squared Error (MSE) and Normalized Root Mean Squared Error (NRMSE). We have included a function in SKM to compute summaries for prediction performance with genomic selection data (summaries). This function requires a by data. frame with whole predictions in different folds, including genotype and environment information; this is used in all the examples described below.

### Wheat Data

This data set was first used by Crossa et al. (2010) and Cuevas et al. (2016), Cuevas et al. (2017) and Cuevas et al. (2019) and is comprised of 599 wheat lines from the CIMMYT Global Wheat Program evaluated in four international environments representing four basic agroclimatic regions (mega-environments). The phenotypic trait considered for the 599 wheat lines evaluated in each of the four mega-environments was grain yield (GY). The 599 wheat lines were genotyped using 1447 Diversity Array Technology (DArT) markers generated by Triticarte Pty. Ltd.

In this example we evaluated the six models included in the package, each one using Bayesian optimization to tune its specific hyperparameters, with the exception of Bayesian methods (model M4), which do not require hyperparameter tuning. The cross-validation used to evaluate the predictions’ accuracy was with five random (splits) partitions, where 80% of the data was used for training and 20% for the testing set, and the average of the five testing sets was reported as prediction performance. To tune the hyperparameters, an inner 5-fold cross validation was also used to evaluate each hyperparameter combination. It is important to point out that the inner 5-fold cross validation is implemented in each partition, which in this case, contains only 80% of the data. In this regard, each inner training contains only 64% of the data while the validation set contains only 16% of the data. In Table 2, the evaluation results are presented for the wheat data set, while the code for implementing the six models is given in Supplementary Appendix SC.

**TABLE 2:** Prediction performance of the Wheat data set for each environment and across environments (Global) of each of the six models.

Model	Metric	E1		E2		E3		E4		Global	
		Mean	SE	Mean	SE	Mean	SE	Mean	SE	Mean	SE
M1	MAAPE	0.7307	0.0069	0.6852	0.0210	0.6993	0.0188	0.6922	0.0104	0.7082	0.0090
M1	MAE	0.6801	0.0133	0.6360	0.0272	0.6644	0.0314	0.5935	0.0162	0.5955	0.0123
M1	MSE	0.7359	0.0210	0.6931	0.0494	0.7908	0.0820	0.6007	0.0332	0.5951	0.0233
M1	NRMSE	0.8575	0.0174	0.8173	0.0263	0.8763	0.0271	0.7915	0.0157	0.8316	0.0081
M1	RMSE	0.8575	0.0123	0.8304	0.0298	0.8839	0.0490	0.7738	0.0221	0.7708	0.0155
M2	MAAPE	0.7134	0.0118	0.7506	0.0107	0.7460	0.0118	0.7635	0.0049	0.7500	0.0112
M2	MAE	0.7023	0.0303	0.7116	0.0292	0.7670	0.0283	0.7179	0.0131	0.6748	0.0163
M2	MSE	0.7845	0.0527	0.8980	0.0646	0.9876	0.0746	0.8581	0.0237	0.7645	0.0289
M2	NRMSE	0.8747	0.0193	0.9429	0.0200	0.9622	0.0145	0.9344	0.0123	0.9311	0.0116
M2	RMSE	0.8836	0.0305	0.9450	0.0349	0.9909	0.0380	0.9260	0.0129	0.8737	0.0167
M3	MAAPE	0.7857	0.0038	0.7835	0.0056	0.7877	0.0010	0.7848	0.0019	0.7856	0.0015
M3	MAE	0.7675	0.0186	0.7972	0.0267	0.7766	0.0082	0.7805	0.0228	0.7341	0.0133
M3	MSE	0.9014	0.0324	1.0875	0.0656	0.9583	0.0268	1.0724	0.0343	0.9035	0.0271
M3	NRMSE	0.9997	0.0013	1.0013	0.0021	1.0012	0.0045	1.0027	0.0017	1.0004	0.0004
M3	RMSE	0.9488	0.0171	1.0409	0.0320	0.9785	0.0138	1.0350	0.0166	0.9501	0.0142
M4	MAAPE	0.7161	0.0134	0.6835	0.0169	0.6902	0.0128	0.6898	0.0204	0.6965	0.0100
M4	MAE	0.6733	0.0273	0.6258	0.0081	0.7060	0.0196	0.5945	0.0094	0.5864	0.0083
M4	MSE	0.7063	0.0450	0.6793	0.0049	0.8221	0.0494	0.6291	0.0409	0.5769	0.0186
M4	NRMSE	0.8472	0.0163	0.8105	0.0159	0.8470	0.0178	0.7963	0.0151	0.8123	0.0080
M4	RMSE	0.8387	0.0264	0.8242	0.0030	0.9050	0.0275	0.7915	0.0252	0.7592	0.0123
M5	MAAPE	0.7133	0.0108	0.6956	0.0107	0.7233	0.0067	0.7455	0.0046	0.7211	0.0043
M5	MAE	0.7141	0.0183	0.6336	0.0116	0.6846	0.0272	0.6572	0.0291	0.6156	0.0056
M5	MSE	0.7987	0.0387	0.6587	0.0170	0.7696	0.0607	0.7021	0.0639	0.6183	0.0104
M5	NRMSE	0.8796	0.0230	0.8168	0.0220	0.8742	0.0121	0.8808	0.0148	0.8547	0.0081
M5	RMSE	0.8927	0.0212	0.8113	0.0104	0.8744	0.0355	0.8346	0.0369	0.7862	0.0066
M6	MAAPE	0.7056	0.0071	0.6991	0.0107	0.7149	0.0132	0.7058	0.0037	0.7075	0.0067
M6	MAE	0.6938	0.0144	0.6358	0.0204	0.6802	0.0280	0.6327	0.0103	0.6170	0.0122
M6	MSE	0.8160	0.0499	0.6978	0.0452	0.7807	0.0678	0.7183	0.0226	0.6645	0.0355
M6	NRMSE	0.8918	0.0067	0.8385	0.0188	0.8889	0.0230	0.8534	0.0167	0.8669	0.0119
M6	RMSE	0.9016	0.0279	0.8336	0.0267	0.8802	0.0386	0.8471	0.0133	0.8140	0.0217

Generalized boosted machines (M1), generalized linear models (M2), support vector machines (M3), random forest (M4), Bayesian regression models (M5) and deep neural networks (M6). The tuning process was done under the Bayesian optimization framework. Mean is the average of the five partitions for each metric and SE denotes the standard error for each metric. E1-E4 denotes location1, location2, location3 and location4.

**FIGURE 1:**
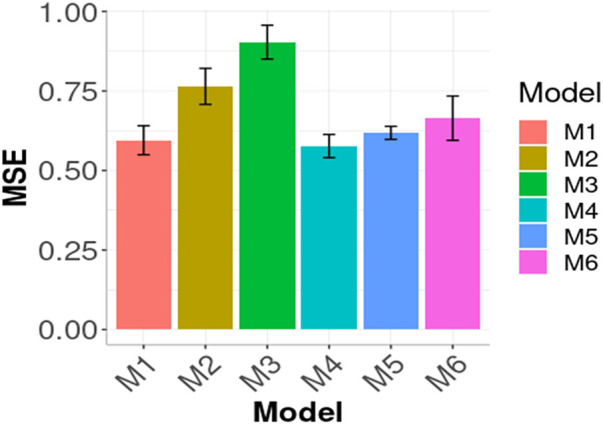
Prediction performance in terms of Mean Squared Error of the six models (M1, M2, M3, M4, M5, M6) across environments (Global) in the wheat data. M1 denotes the generalized boosted machine model, M2 denotes the generalized linear model, M3 denotes the support vector machine model, M4 denotes the random forest model, M5 denotes the Bayesian regression model and M6 denotes the deep neural networks model.

**FIGURE 2:**
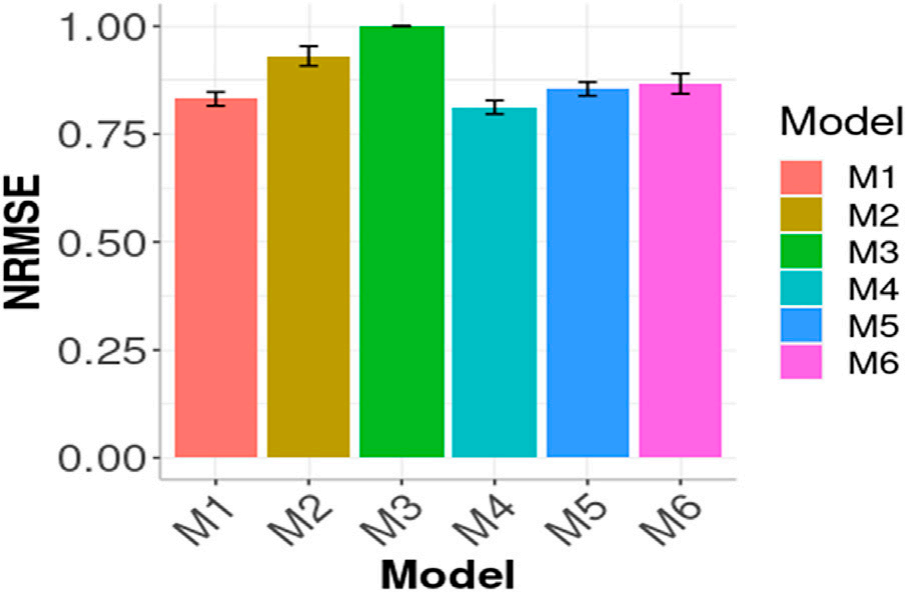
Prediction performance in terms of Normalized Root Mean Squared Error of the six models (M1, M2, M3, M4, M5, M6) across environments (Global) in the wheat data. M1 denotes the generalized boosted machine model, M2 denotes the generalized linear model, M3 denotes the support vector machine model, M4 denotes the random forest model, M5 denotes the Bayesian regression model and M6 denotes the deep neural networks model.

In Figures 1, 2 we compare the prediction performance of the six evaluated models across environments in terms of MSE and NRMSE, respectively. Both figures show a similar pattern in the prediction performance results. In terms of both metrics, M4, M1 and M5 produced the best prediction performance. In terms of MSE, the best model (M4) outperformed M1 by $\left(0.5951-0.5769\right)\times \frac{100}{0.5951}=3.05\%$ , M2 by $\left(0.7645-0.5769\right)\times \frac{100}{0.7645}=32.51\%$ , M3 (the worst) by $\left(0.9035-0.5769\right)\times \frac{100}{0.9035}=36.14\%$ , M5 by $\left(0.6183-0.5769\right)\times \frac{100}{0.6183}=6.69\%$ and M6 by $\left(0.6645-0.5769\right)\times \frac{100}{0.6645}=13.18\%$ . Regarding NRMSE, the outperformance between models is not as large as in MSE terms. For example, the outperformance between the best (M4) and worst (M3) was $\left(1-0.8123\right)\times \frac{100}{1}=18.77\%$ , significantly different from the 36.14% in MSE terms. It should be noted that the model M5 was implemented in all the examples provided with Bayesian Ridge Regression (BRR; that works with the scaled matrix of markers Z ), which is equivalent to BGBLUP [that works with the linear kernel computed as $ZZ^{T}/ncol\left(Z\right)$ ]. As mentioned before, the other Bayesian methods can be implemented by merely changing “BRR” in model to the other available options like: Bayes_A, Bayes_B, Bayes_C, Bayes_Lasso and BGLUP (See Supplementary Appendix SB5. Bayesian regression model).

### Maize Data

This maize data set was included in Souza et al. (2017) and comes from USP (Universidad Sao Paulo). It consists of 722 (with 722 ×4 = 2888 observations) maize hybrids obtained by crossing 49 inbred lines. The hybrids were evaluated in four environments (E1-E4) in Piracicaba and Anhumas, São Paulo, Brazil, in 2016. The hybrids were evaluated using an augmented block design with two commercial hybrids as checks to correct for micro-environmental variation. At each site, two levels of nitrogen (N) fertilization were used. The experiment conducted under ideal N conditions received 100 kg ha-1 of N (30 kg ha-1 at sowing and 70 kg ha-1 in a coverage application) at the V8 plant stage, while the experiment with low N received 30 kg/ha at sowing. The parent lines were genotyped with an Affymetrix Axiom Maize Genotyping Array of 616 K SNPs. Markers with Minor Allele Frequency (MAF) of 0.05 were removed. After applying QC, 54,113 SNPs were available to make the predictions.

In this second example, we evaluated the same cases as the wheat data example using the grid search as a tuning strategy for the hyperparameters. Likewise, in this data set, the prediction performance was evaluated with five random partitions where 80% of the data was used for training and 20% for the testing set and the average of the five testing sets was reported as prediction performance. To tune the hyperparameters, an inner 5-fold cross validation was also used to evaluate each hyperparameter combination. In Table 3 the evaluation results are shown for this data set (Maize). The complete R code for implementing the six models in the SKM library is provided in Supplementary Appendix SD.

**TABLE 3:** Prediction performance of the Maize data set for each environment and across environments (Global) of each of the six models.

Model	Metric	E1		E2		E3		E4		Global	
		Mean	SE	Mean	SE	Mean	SE	Mean	SE	Mean	SE
M1	MAE	0.2038	0.0024	0.4360	0.0122	0.2708	0.0035	0.5392	0.0088	0.3409	0.0047
M1	MSE	0.0700	0.0028	0.2991	0.0179	0.1125	0.0034	0.4670	0.0177	0.2059	0.0059
M1	NRMSE	0.8751	0.0259	0.9021	0.0077	0.9159	0.0135	0.9146	0.0196	0.8872	0.0147
M1	RMSE	0.2644	0.0051	0.5459	0.0166	0.3352	0.0050	0.6829	0.0129	0.4535	0.0066
M2	MAAPE	0.7672	0.0106	0.7787	0.0120	0.7734	0.0102	0.7580	0.0075	0.7565	0.0064
M2	MAE	0.2040	0.0067	0.4687	0.0144	0.2700	0.0060	0.5751	0.0166	0.3592	0.0072
M2	MSE	0.0713	0.0045	0.3460	0.0157	0.1131	0.0064	0.5281	0.0319	0.2336	0.0126
M2	NRMSE	0.9174	0.0119	0.9687	0.0057	0.9353	0.0163	0.9517	0.0070	0.9498	0.0040
M2	RMSE	0.2664	0.0083	0.5876	0.0134	0.3358	0.0095	0.7253	0.0225	0.4826	0.0127
M3	MAAPE	0.7861	0.0059	0.7829	0.0010	0.7871	0.0031	0.7870	0.0023	0.7852	0.0015
M3	MAE	0.2187	0.0054	0.4814	0.0130	0.2855	0.0049	0.6109	0.0151	0.3817	0.0069
M3	MSE	0.0847	0.0034	0.3701	0.0144	0.1287	0.0051	0.5861	0.0325	0.2603	0.0131
M3	NRMSE	1.0023	0.0027	1.0024	0.0031	0.9985	0.0010	1.0032	0.0013	1.0029	0.0014
M3	RMSE	0.2908	0.0059	0.6079	0.0119	0.3584	0.0070	0.7643	0.0215	0.5095	0.0126
M4	MAAPE	0.7450	0.0146	0.7615	0.0114	0.7432	0.0150	0.7418	0.0077	0.7444	0.0063
M4	MAE	0.2006	0.0053	0.4430	0.0100	0.2615	0.0069	0.5586	0.0119	0.3498	0.0034
M4	MSE	0.0678	0.0048	0.3073	0.0136	0.1070	0.0062	0.5041	0.0223	0.2215	0.0054
M4	NRMSE	0.8882	0.0082	0.9320	0.0076	0.9032	0.0173	0.9052	0.0076	0.8997	0.0042
M4	RMSE	0.2598	0.0091	0.5538	0.0122	0.3265	0.0096	0.7093	0.0157	0.4705	0.0058
M5	MAAPE	0.7853	0.0067	0.7601	0.0125	0.7600	0.0064	0.7275	0.0067	0.7483	0.0058
M5	MAE	0.2199	0.0033	0.4507	0.0074	0.2747	0.0086	0.5259	0.0089	0.3426	0.0060
M5	MSE	0.0796	0.0037	0.3269	0.0104	0.1166	0.0067	0.4500	0.0153	0.2099	0.0081
M5	NRMSE	0.9858	0.0087	0.9364	0.0111	0.9533	0.0223	0.8808	0.0063	0.9116	0.0065
M5	RMSE	0.2819	0.0065	0.5714	0.0091	0.3408	0.0100	0.6705	0.0113	0.4578	0.0089
M6	MAAPE	0.7980	0.0126	0.7792	0.0101	0.7819	0.0189	0.7681	0.0113	0.7747	0.0110
M6	MAE	0.2177	0.0075	0.4843	0.0157	0.2907	0.0094	0.5653	0.0200	0.3655	0.0112
M6	MSE	0.0798	0.0048	0.3775	0.0220	0.1398	0.0071	0.4992	0.0290	0.2396	0.0148
M6	NRMSE	0.9720	0.0214	1.0107	0.0114	1.0406	0.0199	0.9267	0.0249	0.9616	0.0215
M6	RMSE	0.2820	0.0084	0.6134	0.0180	0.3735	0.0096	0.7053	0.0212	0.4885	0.0155

Generalized boosted machines (M1), generalized linear models (M2), support vector machines (M3), random forest (M4), Bayesian regression models (M5) and deep neural networks (M6). The tuning process was done under the grid search framework. Mean is the average of the five partitions for each metric, SE denotes the standard error for each metric.

**FIGURE 3:**
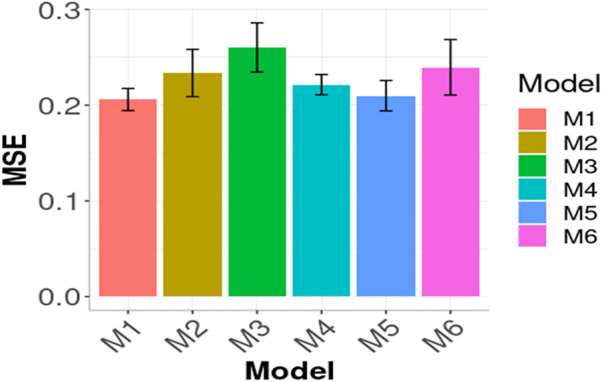
Prediction performance in terms of Mean Squared Error of prediction of the six models (M1, M2, M3, M4, M5, M6) across environment (Global) in Maize data. M1 denotes the generalized boosted machine model, M2 denotes the generalized linear model, M3 denotes the support vector machine model, M4 denotes the random forest model, M5 denotes the Bayesian regression model and M6 denotes the deep neural networks model.

**FIGURE 4:**
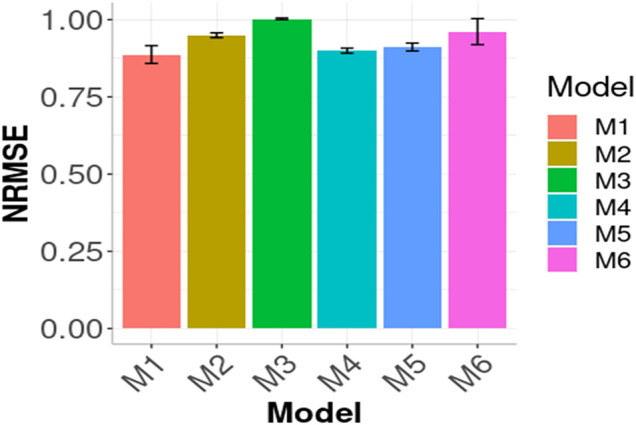
Prediction performance in terms of Normalized Root Mean Squared Error of the six models (M1, M2, M3, M4, M5, M6) across environments (Global) in Maize data. M1 denotes the generalized boosted machine model, M2 denotes the generalized linear model, M3 denotes the support vector machine model, M4 denotes the random forest model, M5 denotes the Bayesian regression model and M6 denotes the deep neural networks model.

In Figures 3, 4 the Global results of the maize data example are presented. Figure 3 shows the prediction performance in terms of MSE and Figure 4 the prediction performance in terms of NRMSE. According to Figure 3, the best Global results were observed in M1 with 0.2059 of MSE followed by M5 0.2099, that is $\left(0.2099-0.2059\right)\times \frac{100}{0.2099}=1.9\%$ worst. M1 outperformed M4 by $\left(0.2215-0.2059\right)\times \frac{100}{0.2215}=7.57\%$ , M2 by $\left(0.2336-0.2059\right)\times \frac{100}{0.2336}=11.85$ %, M6 by $\left(0.2396-0.2059\right)\times \frac{100}{0.2396}=16.36\%$ and M3 (the worst) by $\left(0.2603-0.2059\right)\times \frac{100}{0.2603}=20.89\%$ . In Figure 4 a similar pattern appears: M1 produced the best results since it has the lowest NRMSE. The only change in the order compared to that observed in Figure 3 is that M4 outperformed M5 in terms of NRMSE. The remaining models’ results agree with Figure 3 given that the following best results in terms of NRMSE were obtained with M2, M3 and M6, respectively.

**FIGURE 5:**
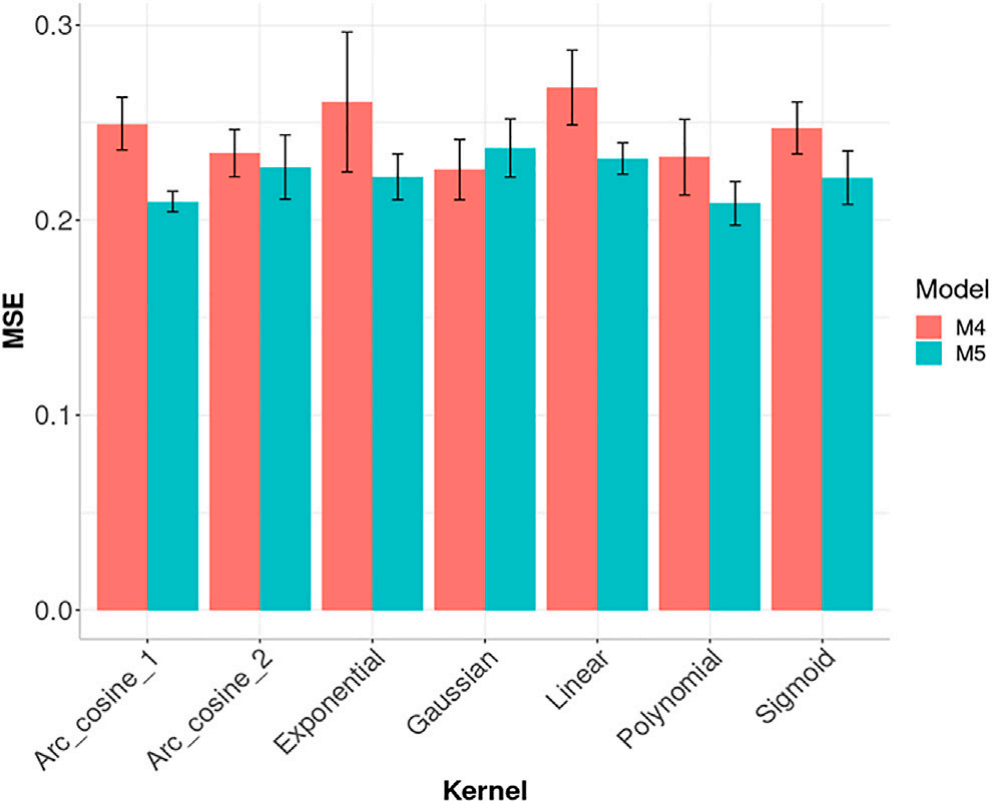
Prediction performance across environments (Global) in Maize data in terms of Mean Square Error (MSE) of models M4 and M5 for seven kernel methods. M4 denotes the random forest model and M5 denotes the Bayesian regression model.

In Figure 5, we compared the performance of seven kernels for the maize data set: Linear, Polynomial, Sigmoid, Gaussian, Exponential, Arc-Cosine_1 and Arc-Cosine_2 for model M4 and M5. For model M5, the best prediction performance was observed under the Arc_cosine_1 and Polynomial kernel and the worst under the Gaussian kernel. While under model M4, the best performance in terms of MSE was observed under the Gaussian Kernel and the worst under the Linear kernel. The code used for implementing model M4 and M5 with the seven kernels are given in Supplementary Appendix SE. It is important to point out that in the SKM library it is possible to perform kernel and sparse kernels not only under the Bayesian BGBLUP method (a sub-model of model M5, that is implemented under a RKHS method in BGLR) but under the six models (M1 to M6) that can be implemented in this library. The kernels apart from one sub-model of model M5 (BGBLUP) are implemented not using as input directly the kernel, but with the square root of the kernel for this reason is possible to be implemented with all the six models. While the sparse kernels were implemented in a similar fashion but using the method explained above, proposed of Cuevas et al. (2020) and for this reason, also it is possible to be implemented with the six models here evaluated (M1, … , M6).

**FIGURE 6:**
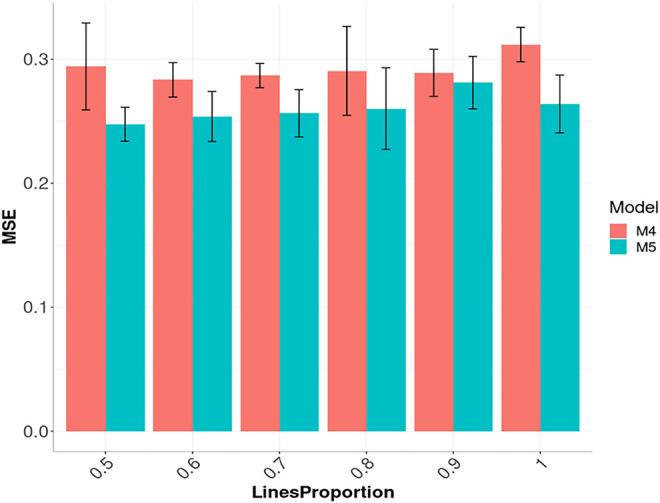
Prediction performance across environments (Global) in Maize data in terms of Mean Square Error (MSE) of models M4 and M5 using the sparse Arc_cosine_1 kernel with six proportions of compression levels: 0.5, 0.4, 0.3, 0.2, 0.1 and 0, which correspond to using only the following proportions: 0.5, 0.6, 0.7, 0.8, 0.9 and 1 of the original lines (LinesProportion) for computing the kernels. M4 denotes the random forest model and M5 denotes the Bayesian regression model. The complement of level of compression level is equal to the proportion of lines used to compute the sparse kernel, that is, level of compression = 1 minus proportion of lines used to compute the sparse kernel.

In Figure 6, we also provide the prediction accuracies in terms of MSE for models M4 and M5 with the Arc_cosine_1 kernel for six compression levels (0.5, 0.4, 0.3, 0.2, 0.1 and 0). It is important to point out that in Figure 6, the complement of the compression levels are given on the *x*-axis, which means the proportion of the columns (subsampling of lines without replacement; see Cuevas et al., 2020) of the complete (dense) kernel that are used as independent variables. We can observe in Figure 6 that the best prediction performance for model M5 was obtained with the compression level at 50%, that is, when the model was trained with only half of the total columns of the complete kernel. However, the worst performance in model M5 was with a compression level of 10% (LinesProportion of 0.9). On the other hand, in model M4, the best and worst prediction performance in terms of MSE was observed under compression level of 0.4 (LinesProportion of 0.6) and 0 (LinesProportion of 1) respectively. The R code for reproducing the results given in Figure 6 are provided in Supplementary Appendix SF.

**FIGURE 7:**
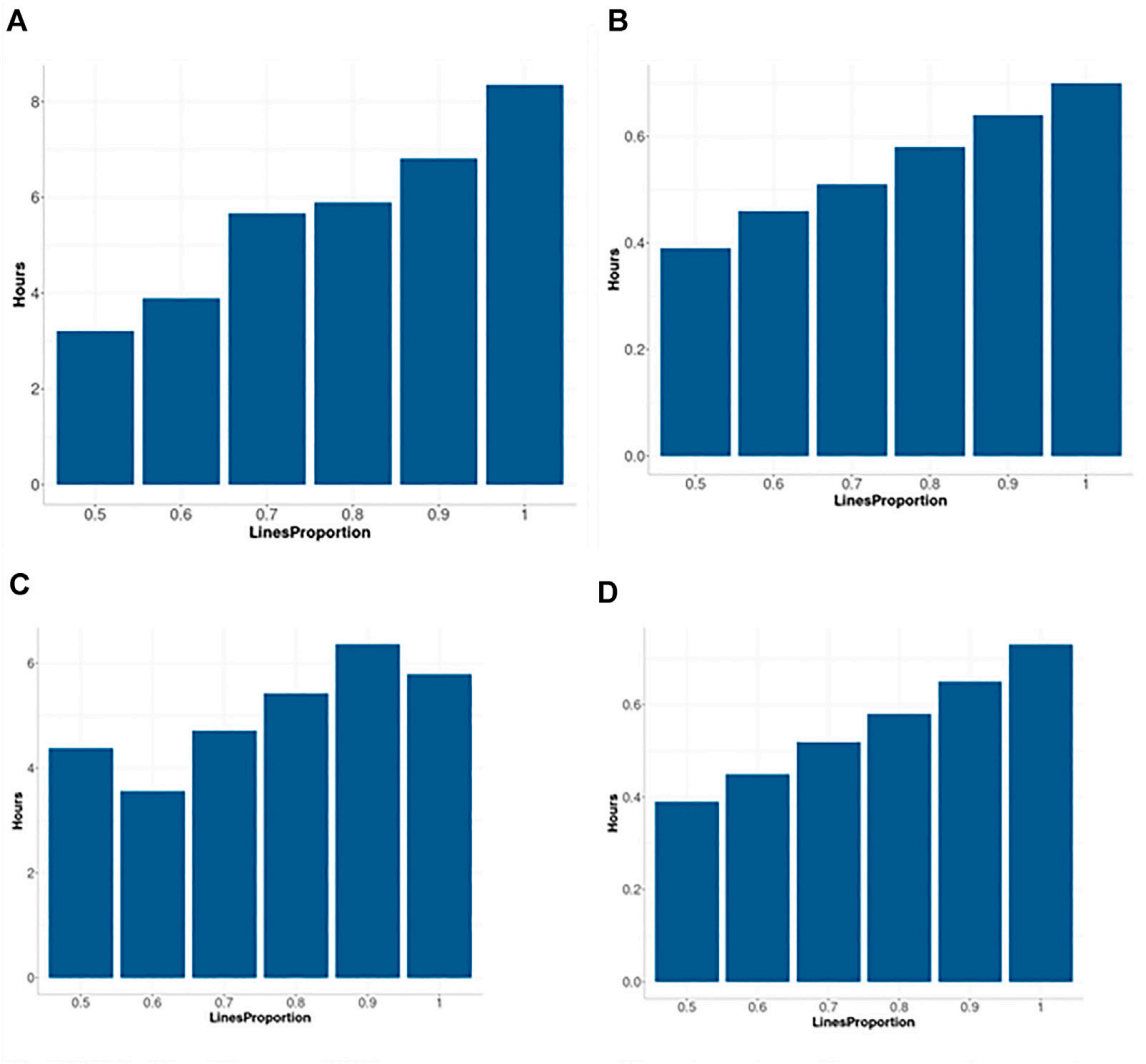
Time in hours for implementing two sparse kernels (Arc_cosine_1 and Gaussian) with the maize data set as a function of the proportion of the compression level (0.5, 0.4, 0.3, 0.2, 0.1 and 0), which corresponds to using only the following proportions: 0.5, 0.6, 0.7, 0.8, 0.9 and 1 of the original lines (LinesProportion) for computing the sparse kernels since level of compression = 1 minus proportion of lines used to compute the sparse kernel. **(A)** corresponds to M4 and Arc_cosine_1 sparse kernel. **(B)** corresponds to M5 Arc_cosine_1 sparse kernel. **(C)** corresponds to M4 and Gaussian sparse kernel. **(D)** corresponds to M5 Gaussian sparse kernel. M4 denotes the random forest model and M5 denotes the Bayesian regression model.

Figure 6 for the Arc_cosine_1 sparse kernel, it is shown that even with the largest compression level, there is not a relevant loss in prediction accuracy. However, when the compression level is larger, less time (in hours) is required for the training process, and the reduction in time of execution is almost linear (Figure 7A for model M4 and Figure 7B for model M5 both for the Arc_cosine_1 sparse kernel). We can also observe in these Figures (Figures 7A,B) that the time required for the training process in model M5 is significantly less than the time required for model M4.

**FIGURE 8:**
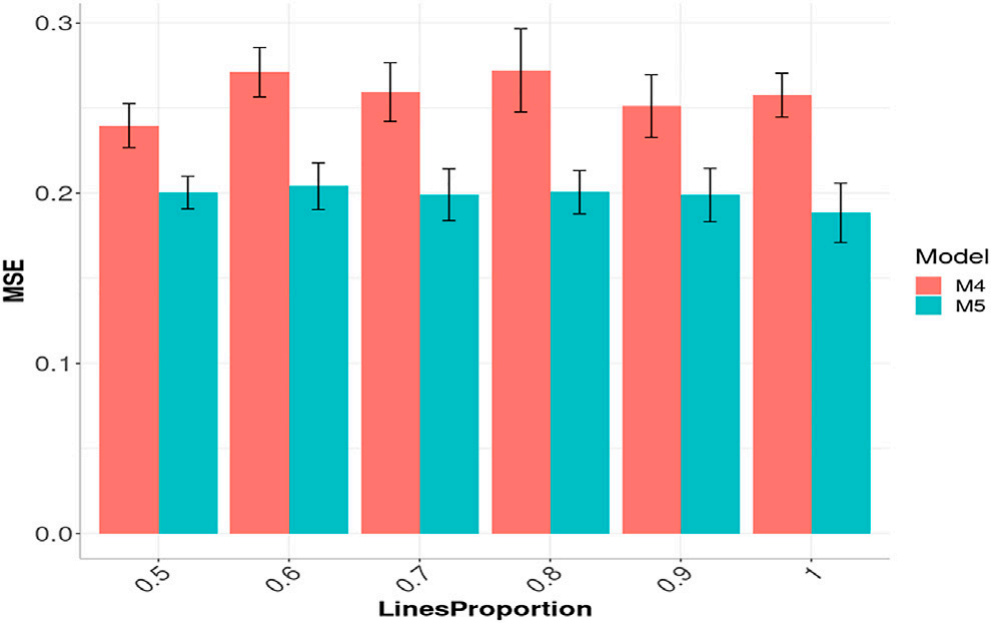
Prediction performance across environments (Global) in Maize data in terms of Mean Square Error (MSE) of models M4 and M5 using the sparse Gaussian kernel with six proportions of compression levels: 0.5, 0.4, 0.3, 0.2, 0.1 and 0, which correspond to using only the following proportions: 0.5, 0.6, 0.7, 0.8, 0.9 and 1 of the original lines (LinesProportion) for computing the sparse kernels, since level of compression = 1 minus proportion of lines used to compute the sparse kernel. M4 denotes the random forest model and M5 denotes the Bayesian regression model.

Figure 8 shows the prediction performance in terms of MSE for models M4 and M5 but now with the Gaussian kernel using the same six compression levels (0.5, 0.4, 0.3, 0.2, 0.1 and 0). For model M5, we did not observe any significant loss in terms of prediction performance with the six levels of compression levels evaluated. In model M5, we can observe that the best prediction performance was obtained with the largest compression level (0.5; LinesProportion of 0.5), but between the remaining compression levels we did not observed significant differences. The R code for reproducing the results given in Figure 8 are provided in Supplementary Appendix SF.

It is observed in Figure 8 that even with the large proportion of compression level, we did not experience a significant loss in prediction accuracy. When the compression level was larger, less time was required for the training process (Figures 7C,D). While the trend is not totally linear under model M4 and Gaussian sparse kernel, it is still clear that a significant reduction in time is achieved when the compression level increases. On the other hand, under model M5 with the Gaussian sparse kernel, a linear reduction is observed in the time required for training when the compression level is increased. This is particularly interesting since we can translate into significant savings of computational resources without a significant loss of prediction accuracy. Furthermore, Figure 7D also shows that model M5 requires considerably less time for the training process in comparison to the model M4 (Figure 7C).

The information provided in this Figure 7, illustrates that with the use of sparse kernels it is possible to gain a significant reduction in time for the implementation of the prediction models by means of dense kernels (without any level of compression). For example, Figure 7 shows that the larger the level of compression the larger the reduction in computational resources. However, as observed in Figures 6, 8 caution must be exercised when determining the level of compression, because when this is large the level of accuracy could be negatively affected (will reduce the prediction performance). However, Figures 6, 8 depicted that even with level of compression of 50% genomic prediction accuracy is not dramatically affected. In general, M4 and M5 with sparse Gaussian kernel enhance the genome-based prediction accuracy of as compared with sparse kernel for all compression levels.

## Default Settings for the Algorithms

The default setting for those algorithms that require a tuning process (M1, M2, M3, M4 and M6) is the “Grid_search” strategy of tuning, but this only works when you specified at least for one of the hyper-parameters with more than two values to be evaluated, that is, a grid with a least two values for at least one hyperparameter. Also, for the tuning process by default is implemented an inner (nested) K-fold cross validation with K = 5 by default. When the Bayesian optimization is selected for the tuning process by default are explored 10 iterations. In Table 4 are given the default hyperparameters for each or the six models.

**TABLE 4:** Default hyper-parameters for each of the models that can be implemented in the SKM library.

Model	Name	Default Hyper-parameter values
M1	Generalized boosted machines	trees_number = 500, max_depth = 1
		node_size = 10, shrinkage = 0.1
		sampled_records_proportion = 0.5
M2	Generalized linear models	alpha = 1; with alpha between 0 and 1 (for Elastic net Regression) and alpha = 0 for Ridge regression and alpha equal to 1 for Lasso Regression
M3	Support vector machines	kernel = “linear”, degree = 3, gamma = 1/NCOL(x), coef0 = 0 and cost = 1
M4	Random forest	trees_number = 500, node_size = 5, node_depth = NULL and sampled_x_vars_number = NULL
M5	Bayesian regression models	Not applied since are not required hyperparameters since run with the default values of the BGLR library
M6	Deep neural networks	learning_rate = 0.001, epochs_number = 500, batch_size = 32, layers = list (list (neurons_number = 50, neurons_proportion = NULL, activation =
		“relu”, dropout = 0, ridge_penalty = 0, lasso_penalty = 0)), output_penalties = list (ridge_penalty = 0, lasso_penalty = 0), optimizer = “adam”, shuffle = TRUE, early_stop = FALSE
		early_stop_patience = 50

## Discussion

Data is playing an unprecedented role in the twenty-first century. For this reason, many companies consider data science as a fundamental component to extract useful knowledge, make better decisions, reduce losses, analyze market trends and increase profits. Likewise, it is playing an essential role in increasing the rate of scientific and technological discoveries. For these reasons, the demand for Data Scientists continues increasing and is expected to grow by 27.9% by 2026, according to the US Bureau of Labor Statistics (Rieley, 2018). However, to satisfy this growing demand, people with different backgrounds need to be trained in this area rather rapidly. In this vein, more open source and user-friendly software such the SKM library are need to extract useful knowledge more efficiently from raw data. Even though there are many tools for implementing supervised machine learning methods in the R statistical software, they are still insufficient to cover the broad spectrum of needs, as there are many complex tasks that are not covered by existing tools.

For example, our library (SKM), in addition to grid search for hyperparameter tuning, also included the Bayesian optimization method, which is a sequential design strategy for global optimization of black-box functions that does not presume any functional forms. It is generally employed to optimize functions that are expensive to evaluate. Bayesian optimization, contrary to a grid search that performs an exhaustive evaluation over each point of the grid of values given for each hyperparameter, needs very few evaluations as starting points, and based on the knowledge at hand, it can indicate which point should be evaluated next. Bayesian optimization makes these decisions with something called acquisition functions, which are heuristics for how desirable it is to evaluate a point based on our present model. At every step, the Bayesian optimization method determines the best point to evaluate according to the acquisition function by optimizing it (Mockus, 2012). The model is then updated, and this process is repeated to determine the next point to evaluate.

In order for machine learning algorithms to be able to successfully perform a grid search, very large amount of values for each hyperparameter is required, and as such, this method is frequently rendered impractical since the required computational resources are substantial. For this reason, our library (SKM) is novel since it can be implemented for hyperparameter tuning with the Bayesian optimization algorithm, which is well suited when the function evaluations are expensive.

We do not expect the proposed SKM library to replace libraries like mlr3 and scikit-learn, since these libraries will continue to be suitable options for those who seek a complete solution for a particular machine learning implementation. Nonetheless, our library (SKM) will be a great alternative for its simplicity, as it can be used with six conventional machine learning algorithms with some kernel methods, and thus, help to better capture non-linear patterns in the data.

Additionally, to the best of our knowledge, this is the first library that permits kernels to be implemented with six conventional machine learning methods in a very simple way, which can help increase the prediction performance when the input data contains non-linear patterns. Furthermore, the SKM package permits the implementation of approximate kernels (here called spare kernels), which can help reduce the computational resources for data sets of large dimensions, without a significant reduction in accuracy. In comparison to typical kernels that reduce the input size to the number of observations, sparse kernels can reduce the input size to even less than the number of observations and in this way, save more computational resources for its implementation. It must be noted that since the building process of the kernels is first done in an independent process, this computed kernel can be implemented with any machine leaning method.

While the proposed SKM library only allows multivariate responses for continuous outcomes to be trained under the Bayesian framework and generalized linear models, it also allows multivariate continuous, binary and categorical outcomes to be trained under by the random forest method. Nevertheless, only deep neural networks allows multivariate responses for continuous, binary, categorical and count to be trained. Contrarily, only univariate models can be trained under generalized boosted machines and support vector machines. As we previously stated, the six models can be implemented with seven kernels. These kernels are Linear, Polynomial, Sigmoid, Gaussian, Exponential, Arc-Cosine 1 and Arc-Cosine L (with L = 2, 3 … ), which is useful for when the dimensionality of the input is larger than the training samples, greater computational resources are needed; however, using any of these kernels reduces the number of training samples which, in turn, reduces the computational resources needed, thus permitting non-linear patterns to be captured more efficiently.

With the illustrative examples provided, the library can implement supervised machine learning methods for binary, categorical, count and continuous response variables, with the advantage that the user does not need to specify the type of response to be implemented; by providing the response variable as a factor, the library will understand whether it will implement a binary or categorical model depending on the number of categories of the response variable. On the other hand, if the response variable is converted to numeric values, the library will implement a count or continuous model.

## Conclusion and Future Work

This new package will benefit both machine learning practitioners and researchers who want to implement predictive models in a simple way with state-of-the art methods for tuning hyperparameters like Bayesian optimization. We also expect people from different disciplines who are not programming experts to be able to take advantage of the simplicity of SKM to enter into the machine learning world.

The kernelize function in SKM is of special interest since this is the first package that allows kernels to be used with different machine learning algorithms as a new approach of working with complex non-linear and high dimensional data.

This new package is not intended to provide a full data science solution, but rather, new machine learning algorithms can be included in future versions along with more metric functions, model benchmarking, data input and other data science related tools.

With the plant breeding examples provided, we illustrated how this library can implement six machine learning algorithms and seven types of kernel methods in the context of genomic prediction. Moreover, we illustrated that the implementation of sparse kernels can save significant computation resources without a significant loss in prediction accuracy. Finally, in the appendices, we provided all the codes so that users from different backgrounds and areas of interest can easily implement all the models and tools provided in the SKM library.

## Data Availability

The original contributions presented in the study are included in the article/Supplementary Material, further inquiries can be directed to the first author and/or the corresponding authors and can be found in: https://github.com/osval78/SKM_Library_Examples.
