# Supplementary material for: A General-Purpose Machine Learning R Library for Sparse Kernels Methods With an Application for Genome-Based Prediction
Source: Front Genet. 2022 Jun 3;13:887643. doi: 10.3389/fgene.2022.887643 (PMC9205295; doi:10.3389/fgene.2022.887643)
Supplement: Supplementary file 1 [file DataSheet1.docx]

# Supplementary Appendix SA

In this section we present the comparative implementation of the same machine learning models using mlr3, SKM and randomForestSRC, the original package. With this example, the benefits of SKM become evident because of its simplicity and code clarity.

We are going to implement a random forest model using the famous iris dataset. This dataset includes the numeric variables Sepal.Length, Sepal.Width, Petal.Length and Petal.Width, and the categorical variable Species. Our model will predict the Sepal.Length variable using as predictors the remaining ones.

## SA1. SKM implementation

**library**(SKM)

**set.seed**(1)

*# Data preparation*
x <- **to_matrix**(iris[, -1])
y <- iris**$**Sepal.Length

*# Create a fold with 80% of data as training and*
*# 20% as testing*
fold <- **cv_random**(
 records_number = **nrow**(x),
 folds_number = 1,
 testing_proportion = 0.2
)
fold <- fold[[1]]

*# Divide data*
x_training <- x[fold**$**training, ]
x_testing <- x[fold**$**testing, ]
y_training <- y[fold**$**training]
y_testing <- y[fold**$**testing]

*# Fit a model using Bayesian optimization for hyperparameter*
*# tuning*
model <- **random_forest**(
 x_training,
 y_training,
 trees_number = **list**(min = 50, max = 200),
 sampled_x_vars_number = **list**(min = 0.3, max = 0.8),
 node_size = **list**(min = 2, max = 20),
 tune_type = "bayesian_optimization",
 tune_bayes_samples_number = 5,
 tune_bayes_iterations_number = 10
)

*# Predict the testing samples*
predictions <- **predict**(model, x_testing)

*# Compute the summary of observed vs predicted*
summary <- **numeric_summary**(y_testing, predictions**$**predicted)
summary
*#> * MSE: 0.1099*
*#> * RMSE: 0.3315*
*#> * NRMSE: 0.3772*
*#> * MAAPE: 0.0488*
*#> * MAE: 0.273*
*#> * Pearson correlation: 0.9253*

## SA2. ml3 implementation

*# If you do not have installed mlr3 packages install them with*
*# the following commands:*
*# install.packages("mlr3verse")*
*# devtools::install_github("mlr-org/mlr3extralearners")*

**library**(mlr3verse)
**library**(mlr3extralearners)
**library**(mlr3tuning)

*# Create the regression task with the iris dataset to predict*
*# the Sepal.Length variable.*
task_iris <- **as_task_regr**(
 iris,
 target = "Sepal.Length",
 id = "iris"
)

*# Create a randomForestSRC based learner.*
learner_iris <- **lrn**("regr.rfsrc")

*# Define the terminator criteria as 15 iterations in tuning*
terminator <- **trm**("evals", n_evals = 15)

*# Define the hyperparameters characteristics*
search_space <- **ps**(
 ntree = **p_int**(lower = 50, upper = 200),
 mtry = **p_int**(lower = 1, upper = 4),
 nodesize = **p_int**(lower = 2, upper = 20)
)

*# Resampling (cross validation) strategy*
hout <- **rsmp**("holdout")
*# Set the loss function to be used during tuning*
measure <- **msr**("regr.mse")

*# Initialize a tuner*
tuner_instance <- TuningInstanceSingleCrit**$new**(
 task = task_iris,
 learner = learner_iris,
 resampling = hout,
 measure = measure,
 search_space = search_space,
 terminator = terminator
)

*# Define the tuner with the tune strategy*
tuner <- **tnr**("grid_search", resolution = 5)
tuner**$optimize**(tuner_instance)

*# Set the best hyperparameters combination to the learner*
learner_iris**$**param_set**$**values <-
 tuner_instance**$**result_learner_param_vals
learner_iris**$train**(task_iris, row_ids = fold**$**training)

predictions <- learner_iris**$predict**(
 task_iris,
 row_ids = fold**$**testing
)

**numeric_summary**(predictions**$**truth, predictions**$**response)
*#> * MSE: 0.1098*
*#> * RMSE: 0.3314*
*#> * NRMSE: 0.3771*
*#> * MAAPE: 0.0484*
*#> * MAE: 0.2704*
*#> * Pearson correlation: 0.9254*

## SA3. randomForestSRC (original package) implementation

**library**(randomForestSRC)

data_training <- iris[fold**$**training, ]
data_testing <- iris[fold**$**testing, ]

model <- **rfsrc**(
 Sepal.Length **~** .,
 data_training,
 ntree = 200,
 mtry = 3,
 nodesize = 5
)
predictions <- **predict**(model, newdata = data_testing)

**numeric_summary**(
 data_testing**$**Sepal.Length,
 predictions**$**predicted
)
*#> * MSE: 0.1094*
*#> * RMSE: 0.3307*
*#> * NRMSE: 0.3763*
*#> * MAAPE: 0.0492*
*#> * MAE: 0.2754*
*#> * Pearson correlation: 0.9256*

Note that all versions implement random forest using the randomForestSRC package in tandem; in the last implementation no tuning is performed.

# Supplementary Appendix SB

In this section we present some examples of how to use the kernelize function in order to compute different kernels and their sparse versions. As the kernelize function receives a *data.frame* or a *matrix* and returns this data after applying the kernel as a matrix, such data can be used in conjunction with the models functions or any other package.

While the following examples will use the iris dataset for simplicity, kernels are recommended for use with high dimensional data.

**library**(SKM)

**set.seed**(2)

y <- iris**$**Species
x_polynomial <- **kernelize**(
 iris[, -5],
 kernel = "polynomial",
 degree = 3
)
*# After kernelize, x is now a square matrix*
**dim**(x_polynomial)

x_sparse_polynomial <- **kernelize**(
 iris[, -5],
 kernel = "sparse_polynomial",
 rows_proportion = 0.6,
 degree = 4
)
*# After the sparse kernel, x has at most as many columns as*
*# 60% rows*
**dim**(x_sparse_polynomial)

## SB1. Generalized boosted machine

*# Using x sparse kernelized to train the model*
model <- **generalized_boosted_machine**(
 x_sparse_polynomial,
 y,
 trees_number = 500,
 max_depth = 15,
 shrinkage = 0.001
)

*# We are going to predict the same samples used*
*# in training*
predictions <- **predict**(model, x_polynomial)

**categorical_summary**(y, predictions**$**predicted)

## SB2. Generalized linear model

*# Using x kernelized to train the model*
model <- **generalized_linear_model**(
 x_polynomial,
 y,
 alpha = 0
)

*# We are going to predict the same samples used*
*# in training*
predictions <- **predict**(model, x_polynomial)

**categorical_summary**(y, predictions**$**predicted)

## SB3. Support vector machine

*# Using x sparse kernelized to train the model*
model <- **support_vector_machine**(
 x_sparse_polynomial,
 y,
 kernel = "polynomial",
 degree = 3,
 gamma = 0.2,
 cost = 1
)

*# We are going to predict the same samples used*
*# in training*
predictions <- **predict**(model, x_polynomial)

**categorical_summary**(y, predictions**$**predicted)

## SB4. Random forest

*# Using x sparse kernelized to train the model*
model <- **random_forest**(
 x_polynomial,
 y,
 trees_number = 500,
 sampled_x_vars_number = 0.4,
 node_size = 5
)

*# We are going to predict the same samples used*
*# in training*
predictions <- **predict**(model, x_polynomial)

**categorical_summary**(y, predictions**$**predicted)

## SB5. Bayesian regression model

*# Using x sparse kernelized to train the model*
model <- **bayesian_model**(
 x = **list**(**list**(
 x = x_sparse_polynomial,
 model = "BRR"
 )),
 y = y,
 iterations_number = 10000,
 burn_in = 5000
)

*# We are going to predict the same samples used*
*# in training*
predictions <- **predict**(model,1:length(y))

**categorical_summary**(y, predictions**$**predicted)

## SB6. Deep neural network

*# Now we are going to fit a deep learning model*
*# using the sparse kernel matrix*
sparse_model <- **deep_learning**(
 x_sparse_polynomial,
 y,
 epochs_number = 100,
 learning_rate = **c**(0.001, 0.01),
 layers = **list**(
 *# First hidden layer*
 **list**(
 neurons_number = **c**(50, 100),
 activation = "relu",
 dropout = 0.2
 ),
 *# Second hidden layer*
 **list**(
 neurons_number = **c**(50, 100),
 activation = "relu",
 dropout = 0.2
 )
 )
)

predictions <- **predict**(sparse_model, x_sparse_polynomial)

**categorical_summary**(
 observed = y,
 predicted = predictions**$**predicted,
 probabilities = predictions**$**probabilities
)

Even though in this example we only use the polynomial kernel and its sparse version, the same applies to all other available kernels; the desired kernel must only be specified.

# Supplementary Appendix SC

In this section we provide the code for implementing the six models: M1) generalized boosted machines M2) generalized linear models M3) support vector machines M4) random forest M5) Bayesian regression models and M6) deep neural networks for the wheat data set using Bayesian optimization. It is important to point out that in the predictor we included the information of Environments, Genotypes and Genotype$\times$Environment interaction.

## SC1. Generalized boosted machine

**rm**(list = **ls**())

**library**(SKM)

data(Wheat)

Pheno=Wheat$Pheno
Geno= Wheat$Geno

*# Data preparation*
Line <- **model.matrix**(**~**0 **+** Line, data = Pheno)
Env <- **model.matrix**(**~**0 **+** Env, data = Pheno)
Geno <- **t**(**chol**(Geno[, -1]))
LinexGeno <- Line **%*%** Geno
LinexGenoxEnv <- **model.matrix**(**~** 0 **+** LinexGeno**:**Env)

X <- **cbind**(Env, LinexGeno, LinexGenoxEnv)
y <- Pheno**$**Y

folds <- **cv_random**(
 records_number = **nrow**(X),
 folds_number = 5,
 testing_proportion = 0.2
)

Predictions <- **data.frame**()

**for** (i **in** **seq_along**(folds)) {
 **cat**("*** Fold:", i, " ***\n")
 fold <- folds[[i]]

 X_training <- X[fold**$**training, ]
 X_testing <- X[fold**$**testing, ]
 y_training <- y[fold**$**training]
 y_testing <- y[fold**$**testing]

 model <- **generalized_boosted_machine**(
 x = X_training,
 y = y_training,

 *# Specify the hyperparameters ranges*
 trees_number = **list**(min = 100, max = 500),
 max_depth = **list**(min = 15, max = 40),
 shrinkage = **list**(min = 0.001, max = 0.1),

 tune_cv_type = "random",
 tune_folds_number = 5,
 tune_testing_proportion = 0.2,

 tune_type = "bayesian_optimization",
 tune_bayes_samples_number = 5,
 tune_bayes_iterations_number = 5
 )

 predictions <- **predict**(model, X_testing)

 FoldPredictions <- **data.frame**(
 Fold = i,
 Line = Pheno**$**Line[fold**$**testing],
 Env = Pheno**$**Env[fold**$**testing],
 Observed = y_testing,
 Predicted = predictions**$**predicted
 )
 Predictions <- **rbind**(Predictions, FoldPredictions)
}

summaries <- **gs_summaries**(
 Predictions,
 save_at = "results/bayesian/M1"
)

## SC2. Generalized linear model

**rm**(list = **ls**())

**library**(SKM)

data(Wheat)

Pheno=Wheat$Pheno
Geno= Wheat$Geno

*# Data preparation*
Line <- **model.matrix**(**~**0 **+** Line, data = Pheno)
Env <- **model.matrix**(**~**0 **+** Env, data = Pheno)
Geno <- **t**(**chol**(Geno[, -1]))
LinexGeno <- Line **%*%** Geno
LinexGenoxEnv <- **model.matrix**(**~** 0 **+** LinexGeno**:**Env)

X <- **cbind**(Env, LinexGeno, LinexGenoxEnv)
y <- Pheno**$**Y

folds <- **cv_random**(
 records_number = **nrow**(X),
 folds_number = 5,
 testing_proportion = 0.2
)

Predictions <- **data.frame**()

**for** (i **in** **seq_along**(folds)) {
 **cat**("*** Fold:", i, " ***\n")
 fold <- folds[[i]]

 X_training <- X[fold**$**training, ]
 X_testing <- X[fold**$**testing, ]
 y_training <- y[fold**$**training]
 y_testing <- y[fold**$**testing]

 model <- **generalized_linear_model**(
 x = X_training,
 y = y_training,

 *# Specify the hyperparameters ranges*
 alpha = **list**(min = 0, max = 1),

 tune_folds_number = 5,

 tune_type = "bayesian_optimization",
 tune_bayes_samples_number = 5,
 tune_bayes_iterations_number = 5
 )

 predictions <- **predict**(model, X_testing)

 FoldPredictions <- **data.frame**(
 Fold = i,
 Line = Pheno**$**Line[fold**$**testing],
 Env = Pheno**$**Env[fold**$**testing],
 Observed = y_testing,
 Predicted = predictions**$**predicted
 )
 Predictions <- **rbind**(Predictions, FoldPredictions)
}

summaries <- **gs_summaries**(
 Predictions,
 save_at = "results/bayesian/M2"
)

## SC3. Support vector machine

**rm**(list = **ls**())

**library**(SKM)

data(Wheat)

Pheno=Wheat$Pheno
Geno= Wheat$Geno

*# Data preparation*
Line <- **model.matrix**(**~**0 **+** Line, data = Pheno)
Env <- **model.matrix**(**~**0 **+** Env, data = Pheno)
Geno <- **t**(**chol**(Geno[, -1]))
LinexGeno <- Line **%*%** Geno
LinexGenoxEnv <- **model.matrix**(**~** 0 **+** LinexGeno**:**Env)

X <- **cbind**(Env, LinexGeno, LinexGenoxEnv)
y <- Pheno**$**Y

folds <- **cv_random**(
 records_number = **nrow**(X),
 folds_number = 5,
 testing_proportion = 0.2
)

Predictions <- **data.frame**()

**for** (i **in** **seq_along**(folds)) {
 **cat**("*** Fold:", i, " ***\n")
 fold <- folds[[i]]

 X_training <- X[fold**$**training, ]
 X_testing <- X[fold**$**testing, ]
 y_training <- y[fold**$**training]
 y_testing <- y[fold**$**testing]

 model <- **support_vector_machine**(
 x = X_training,
 y = y_training,

 kernel = "radial",
 *# Specify the hyperparameters ranges*
 gamma = **list**(min = 0.2, max = 0.9),
 cost = **list**(min = 0.5, max = 1),

 tune_cv_type = "random",
 tune_folds_number = 5,
 tune_testing_proportion = 0.2,

 tune_type = "bayesian_optimization",
 tune_bayes_samples_number = 5,
 tune_bayes_iterations_number = 5
 )

 predictions <- **predict**(model, X_testing)

 FoldPredictions <- **data.frame**(
 Fold = i,
 Line = Pheno**$**Line[fold**$**testing],
 Env = Pheno**$**Env[fold**$**testing],
 Observed = y_testing,
 Predicted = predictions**$**predicted
 )
 Predictions <- **rbind**(Predictions, FoldPredictions)
}

summaries <- **gs_summaries**(
 Predictions,
 save_at = "results/bayesian/M3"
)

## SC4. Random forest

**rm**(list = **ls**())

**library**(SKM)

data(Wheat)

Pheno=Wheat$Pheno
Geno= Wheat$Geno

*# Data preparation*
Line <- **model.matrix**(**~**0 **+** Line, data = Pheno)
Env <- **model.matrix**(**~**0 **+** Env, data = Pheno)
Geno <- **t**(**chol**(Geno[, -1]))
LinexGeno <- Line **%*%** Geno
LinexGenoxEnv <- **model.matrix**(**~** 0 **+** LinexGeno**:**Env)

X <- **cbind**(Env, LinexGeno, LinexGenoxEnv)
y <- Pheno**$**Y

folds <- **cv_random**(
 records_number = **nrow**(X),
 folds_number = 5,
 testing_proportion = 0.2
)

Predictions <- **data.frame**()

**for** (i **in** **seq_along**(folds)) {
 **cat**("*** Fold:", i, " ***\n")
 fold <- folds[[i]]

 X_training <- X[fold**$**training, ]
 X_testing <- X[fold**$**testing, ]
 y_training <- y[fold**$**training]
 y_testing <- y[fold**$**testing]

 model <- **random_forest**(
 x = X_training,
 y = y_training,

 *# Specify the hyperparameters ranges*
 trees_number = **list**(min = 100, max = 500),
 sampled_x_vars_number = **list**(min = 0.3, max = 0.8),
 node_size = **list**(min = 2, max = 15),

 tune_cv_type = "random",
 tune_folds_number = 5,
 tune_testing_proportion = 0.2,

 tune_type = "bayesian_optimization",
 tune_bayes_samples_number = 5,
 tune_bayes_iterations_number = 5
 )

 predictions <- **predict**(model, X_testing)

 FoldPredictions <- **data.frame**(
 Fold = i,
 Line = Pheno**$**Line[fold**$**testing],
 Env = Pheno**$**Env[fold**$**testing],
 Observed = y_testing,
 Predicted = predictions**$**predicted
 )
 Predictions <- **rbind**(Predictions, FoldPredictions)
}

summaries <- **gs_summaries**(
 Predictions,
 save_at = "results/bayesian/M4"
)

## SC5. Bayesian regression model

**rm**(list = **ls**())

**library**(SKM)

data(Wheat)

Pheno=Wheat$Pheno
Geno= Wheat$Geno

*# Data preparation*
Line <- **model.matrix**(**~**0 **+** Line, data = Pheno)
Env <- **model.matrix**(**~**0 **+** Env, data = Pheno)
Geno <- **t**(**chol**(Geno[, -1]))
LinexGeno <- Line **%*%** Geno
LinexGenoxEnv <- **model.matrix**(**~** 0 **+** LinexGeno**:**Env)

X <- **list**(
 Env = **list**(x = Env, model = "FIXED"),
 LinexGeno = **list**(x = LinexGeno, model = "BRR"),
 LinexGenoxEnv = **list**(x = LinexGenoxEnv, model = "BRR")
)

y <- Pheno**$**Y

folds <- **cv_random**(
 records_number = **length**(y),
 folds_number = 5,
 testing_proportion = 0.2
)

Predictions <- **data.frame**()

**for** (i **in** **seq_along**(folds)) {
 **cat**("*** Fold:", i, " ***\n")
 fold <- folds[[i]]

 *# This function receives the whole data and the*
 *# testing indices separately*
 model <- **bayesian_model**(
 x = X,
 y = y,

 testing_indices = fold**$**testing,

 iterations_number = 10000,
 burn_in = 5000
 )

 *# Extract the predicted values of testing*
 predictions <- **predict**(model)

 FoldPredictions <- **data.frame**(
 Fold = i,
 Line = Pheno**$**Line[fold**$**testing],
 Env = Pheno**$**Env[fold**$**testing],
 Observed = y[fold**$**testing],
 Predicted = predictions**$**predicted
 )
 Predictions <- **rbind**(Predictions, FoldPredictions)
}

summaries <- **gs_summaries**(
 Predictions,
 save_at = "results/bayesian/M5"
)

## SC6. Deep neural network

**rm**(list = **ls**())

**library**(SKM)

data(Wheat)

Pheno=Wheat$Pheno
Geno= Wheat$Geno

*# Data preparation*
Line <- **model.matrix**(**~**0 **+** Line, data = Pheno)
Env <- **model.matrix**(**~**0 **+** Env, data = Pheno)
Geno <- **t**(**chol**(Geno[, -1]))
LinexGeno <- Line **%*%** Geno
LinexGenoxEnv <- **model.matrix**(**~** 0 **+** LinexGeno**:**Env)

X <- **cbind**(Env, LinexGeno, LinexGenoxEnv)
y <- Pheno**$**Y

folds <- **cv_random**(
 records_number = **nrow**(X),
 folds_number = 5,
 testing_proportion = 0.2
)

Predictions <- **data.frame**()

**for** (i **in** **seq_along**(folds)) {
 **cat**("*** Fold:", i, " ***\n")
 fold <- folds[[i]]

 X_training <- X[fold**$**training, ]
 X_testing <- X[fold**$**testing, ]
 y_training <- y[fold**$**training]
 y_testing <- y[fold**$**testing]

 model <- **deep_learning**(
 x = X_training,
 y = y_training,

 *# Specify the hyperparameters ranges*
 epochs_number = **list**(min = 100, max = 500),
 learning_rate = **list**(min = 0.001, max = 0.1),
 layers = **list**(
 *# First hidden layer*
 **list**(
 neurons_number = **list**(min = 50, max = 150),
 activation = "relu",
 dropout = **list**(min = 0.1, max = 0.5)
 ),
 *# Second hidden layer*
 **list**(
 neurons_number = **list**(min = 50, max = 150),
 activation = "relu",
 dropout = **list**(min = 0.1, max = 0.5)
 )
 ),

 tune_cv_type = "random",
 tune_folds_number = 5,
 tune_testing_proportion = 0.2,

 tune_type = "bayesian_optimization",
 tune_bayes_samples_number = 5,
 tune_bayes_iterations_number = 5
 )

 predictions <- **predict**(model, X_testing)

 FoldPredictions <- **data.frame**(
 Fold = i,
 Line = Pheno**$**Line[fold**$**testing],
 Env = Pheno**$**Env[fold**$**testing],
 Observed = y_testing,
 Predicted = predictions**$**predicted
 )
 Predictions <- **rbind**(Predictions, FoldPredictions)
}

summaries <- **gs_summaries**(
 Predictions,
 save_at = "results/bayesian/M6"
)

# Supplementary Appendix SD

In this section we provide the code for implementing the six models: M1) generalized boosted machines M2) generalized linear models M3) support vector machines M4) random forest M5) Bayesian regression models and M6) deep neural networks for the maize data set using a grid search. It should be highlighted that in the predictor, we included the information of Environments, Genotypes and Genotype$\times$Environment interaction.

## SD1. Generalized boosted machine

**rm**(list = **ls**())

**library**(SKM)

data(Maize)

Pheno= Maize$Pheno
Geno= Maize$Geno

*# Data preparation*
Line <- **model.matrix**(**~**0 **+** Line, data = Pheno)
Env <- **model.matrix**(**~**0 **+** Env, data = Pheno)
Geno <- BMTME**::cholesky**(Geno[, -1])
LinexGeno <- Line **%*%** Geno
LinexGenoxEnv <- **model.matrix**(**~** 0 **+** LinexGeno**:**Env)

X <- **cbind**(Env, LinexGeno, LinexGenoxEnv)
y <- Pheno**$**Y

folds <- **cv_random**(
 records_number = **nrow**(X),
 folds_number = 5,
 testing_proportion = 0.2
)

Predictions <- **data.frame**()

**for** (i **in** **seq_along**(folds)) {
 **cat**("*** Fold:", i, " ***\n")
 fold <- folds[[i]]

 X_training <- X[fold**$**training, ]
 X_testing <- X[fold**$**testing, ]
 y_training <- y[fold**$**training]
 y_testing <- y[fold**$**testing]

 model <- **generalized_boosted_machine**(
 x = X_training,
 y = y_training,

 *# Specify the hyperparameters values*
 trees_number = **c**(100, 300, 500),
 max_depth = **c**(5, 10, 15),
 shrinkage = **c**(0.001, 0.01, 0.1),

 tune_cv_type = "random",
 tune_folds_number = 5,
 tune_testing_proportion = 0.2,

 tune_type = "grid_search",
 tune_grid_proportion = 0.5
 )

 predictions <- **predict**(model, X_testing)

 FoldPredictions <- **data.frame**(
 Fold = i,
 Line = Pheno**$**Line[fold**$**testing],
 Env = Pheno**$**Env[fold**$**testing],
 Observed = y_testing,
 Predicted = predictions**$**predicted
 )
 Predictions <- **rbind**(Predictions, FoldPredictions)
}

summaries <- **gs_summaries**(
 Predictions,
 save_at = "results/grid/M1"
)

## SD2. Generalized linear model

**rm**(list = **ls**())

**library**(SKM)

data(Maize)

Pheno= Maize$Pheno
Geno= Maize$Geno

*# Data preparation*
Line <- **model.matrix**(**~**0 **+** Line, data = Pheno)
Env <- **model.matrix**(**~**0 **+** Env, data = Pheno)
Geno <- BMTME**::cholesky**(Geno[, -1])
LinexGeno <- Line **%*%** Geno
LinexGenoxEnv <- **model.matrix**(**~** 0 **+** LinexGeno**:**Env)

X <- **cbind**(Env, LinexGeno, LinexGenoxEnv)
y <- Pheno**$**Y

folds <- **cv_random**(
 records_number = **nrow**(X),
 folds_number = 5,
 testing_proportion = 0.2
)

Predictions <- **data.frame**()

**for** (i **in** **seq_along**(folds)) {
 **cat**("*** Fold:", i, " ***\n")
 fold <- folds[[i]]

 X_training <- X[fold**$**training, ]
 X_testing <- X[fold**$**testing, ]
 y_training <- y[fold**$**training]
 y_testing <- y[fold**$**testing]

 model <- **generalized_linear_model**(
 x = X_training,
 y = y_training,

 *# Specify the hyperparameters values*
 alpha = **c**(0, 0.4, 0.8, 1),

 tune_folds_number = 5,

 tune_type = "grid_search",
 tune_grid_proportion = 1
 )

 predictions <- **predict**(model, X_testing)

 FoldPredictions <- **data.frame**(
 Fold = i,
 Line = Pheno**$**Line[fold**$**testing],
 Env = Pheno**$**Env[fold**$**testing],
 Observed = y_testing,
 Predicted = predictions**$**predicted
 )
 Predictions <- **rbind**(Predictions, FoldPredictions)
}

summaries <- **gs_summaries**(
 Predictions,
 save_at = "results/grid/M2"
)

## SD3. Support vector machine

**rm**(list = **ls**())

**library**(SKM)

data(Maize)

Pheno= Maize$Pheno
Geno= Maize$Geno

*# Data preparation*
Line <- **model.matrix**(**~**0 **+** Line, data = Pheno)
Env <- **model.matrix**(**~**0 **+** Env, data = Pheno)
Geno <- BMTME**::cholesky**(Geno[, -1])
LinexGeno <- Line **%*%** Geno
LinexGenoxEnv <- **model.matrix**(**~** 0 **+** LinexGeno**:**Env)

X <- **cbind**(Env, LinexGeno, LinexGenoxEnv)
y <- Pheno**$**Y

folds <- **cv_random**(
 records_number = **nrow**(X),
 folds_number = 5,
 testing_proportion = 0.2
)

Predictions <- **data.frame**()

**for** (i **in** **seq_along**(folds)) {
 **cat**("*** Fold:", i, " ***\n")
 fold <- folds[[i]]

 X_training <- X[fold**$**training, ]
 X_testing <- X[fold**$**testing, ]
 y_training <- y[fold**$**training]
 y_testing <- y[fold**$**testing]

 model <- **support_vector_machine**(
 x = X_training,
 y = y_training,

 kernel = "radial",
 *# Specify the hyperparameters values*
 gamma = **c**(0.2, 0.5, 0.9),
 cost = **c**(0.5, 0.8, 1),

 tune_cv_type = "random",
 tune_folds_number = 5,
 tune_testing_proportion = 0.2,

 tune_type = "grid_search",
 tune_grid_proportion = 1
 )

 predictions <- **predict**(model, X_testing)

 FoldPredictions <- **data.frame**(
 Fold = i,
 Line = Pheno**$**Line[fold**$**testing],
 Env = Pheno**$**Env[fold**$**testing],
 Observed = y_testing,
 Predicted = predictions**$**predicted
 )
 Predictions <- **rbind**(Predictions, FoldPredictions)
}

summaries <- **gs_summaries**(
 Predictions,
 save_at = "results/grid/M3"
)

## SD4. Random forest

**rm**(list = **ls**())

**library**(SKM)

data(Maize)

Pheno= Maize$Pheno
Geno= Maize$Geno

*# Data preparation*
Line <- **model.matrix**(**~**0 **+** Line, data = Pheno)
Env <- **model.matrix**(**~**0 **+** Env, data = Pheno)
Geno <- BMTME**::cholesky**(Geno[, -1])
LinexGeno <- Line **%*%** Geno
LinexGenoxEnv <- **model.matrix**(**~** 0 **+** LinexGeno**:**Env)

X <- **cbind**(Env, LinexGeno, LinexGenoxEnv)
y <- Pheno**$**Y

folds <- **cv_random**(
 records_number = **nrow**(X),
 folds_number = 5,
 testing_proportion = 0.2
)

Predictions <- **data.frame**()

**for** (i **in** **seq_along**(folds)) {
 **cat**("*** Fold:", i, " ***\n")
 fold <- folds[[i]]

 X_training <- X[fold**$**training, ]
 X_testing <- X[fold**$**testing, ]
 y_training <- y[fold**$**training]
 y_testing <- y[fold**$**testing]

 model <- **random_forest**(
 x = X_training,
 y = y_training,

 *# Specify the hyperparameters values*
 trees_number = **c**(100, 300, 500),
 sampled_x_vars_number = **c**(0.3, 0.5, 0.8),
 node_size = **c**(5, 10),

 tune_cv_type = "random",
 tune_folds_number = 5,
 tune_testing_proportion = 0.2,

 tune_type = "grid_search",
 tune_grid_proportion = 0.8
 )

 predictions <- **predict**(model, X_testing)

 FoldPredictions <- **data.frame**(
 Fold = i,
 Line = Pheno**$**Line[fold**$**testing],
 Env = Pheno**$**Env[fold**$**testing],
 Observed = y_testing,
 Predicted = predictions**$**predicted
 )
 Predictions <- **rbind**(Predictions, FoldPredictions)
}

summaries <- **gs_summaries**(
 Predictions,
 save_at = "results/grid/M4"
)

## SD5. Bayesian regression model

**rm**(list = **ls**())

**library**(SKM)

data(Maize)

Pheno= Maize$Pheno
Geno= Maize$Geno

*# Data preparation*
Line <- **model.matrix**(**~**0 **+** Line, data = Pheno)
Env <- **model.matrix**(**~**0 **+** Env, data = Pheno)
Geno <- BMTME**::cholesky**(Geno[, -1])
LinexGeno <- Line **%*%** Geno
LinexGenoxEnv <- **model.matrix**(**~** 0 **+** LinexGeno**:**Env)

X <- **list**(
 Env = **list**(x = Env, model = "FIXED"),
 LinexGeno = **list**(x = LinexGeno, model = "BRR"),
 LinexGenoxEnv = **list**(x = LinexGenoxEnv, model = "BRR")
)

y <- Pheno**$**Y

folds <- **cv_random**(
 records_number = **length**(y),
 folds_number = 5,
 testing_proportion = 0.2
)

Predictions <- **data.frame**()

**for** (i **in** **seq_along**(folds)) {
 **cat**("*** Fold:", i, " ***\n")
 fold <- folds[[i]]

 *# This function receives the whole data and the*
 *# testing indices separately*
 model <- **bayesian_model**(
 x = X,
 y = y,

 testing_indices = fold**$**testing,

 iterations_number = 10000,
 burn_in = 5000
 )

 *# Extract the predicted values of testing*
 predictions <- **predict**(model)

 FoldPredictions <- **data.frame**(
 Fold = i,
 Line = Pheno**$**Line[fold**$**testing],
 Env = Pheno**$**Env[fold**$**testing],
 Observed = y[fold**$**testing],
 Predicted = predictions**$**predicted
 )
 Predictions <- **rbind**(Predictions, FoldPredictions)
}

summaries <- **gs_summaries**(
 Predictions,
 save_at = "results/grid/M5"
)

## SD6. Deep neural network

**rm**(list = **ls**())

**library**(SKM)

data(Maize)

Pheno= Maize$Pheno
Geno= Maize$Geno

*# Data preparation*
Line <- **model.matrix**(**~**0 **+** Line, data = Pheno)
Env <- **model.matrix**(**~**0 **+** Env, data = Pheno)
Geno <- BMTME**::cholesky**(Geno[, -1])
LinexGeno <- Line **%*%** Geno
LinexGenoxEnv <- **model.matrix**(**~** 0 **+** LinexGeno**:**Env)

X <- **cbind**(Env, LinexGeno, LinexGenoxEnv)
y <- Pheno**$**Y

folds <- **cv_random**(
 records_number = **nrow**(X),
 folds_number = 5,
 testing_proportion = 0.2
)

Predictions <- **data.frame**()

**for** (i **in** **seq_along**(folds)) {
 **cat**("*** Fold:", i, " ***\n")
 fold <- folds[[i]]

 X_training <- X[fold**$**training, ]
 X_testing <- X[fold**$**testing, ]
 y_training <- y[fold**$**training]
 y_testing <- y[fold**$**testing]

 model <- **deep_learning**(
 x = X_training,
 y = y_training,

 *# Specify the hyperparameters values*
 epochs_number = **c**(100, 300),
 learning_rate = **c**(0.001, 0.1),
 layers = **list**(
 *# First hidden layer*
 **list**(
 neurons_number = **c**(50, 100),
 activation = "relu"
 ),
 *# Second hidden layer*
 **list**(
 neurons_number = 100,
 activation = **c**("relu", "sigmoid"),
 dropout = 0.2
 )
 ),

 tune_cv_type = "random",
 tune_folds_number = 5,
 tune_testing_proportion = 0.2,

 tune_type = "grid_search",
 tune_grid_proportion = 0.8
 )

 predictions <- **predict**(model, X_testing)

 FoldPredictions <- **data.frame**(
 Fold = i,
 Line = Pheno**$**Line[fold**$**testing],
 Env = Pheno**$**Env[fold**$**testing],
 Observed = y_testing,
 Predicted = predictions**$**predicted
 )
 Predictions <- **rbind**(Predictions, FoldPredictions)
}

summaries <- **gs_summaries**(
 Predictions,
 save_at = "results/grid/M6"
)

# Supplementary Appendix SE

In this section we provide the code for implementing seven kernel models: Linear, Polynomial, Sigmoid, Gaussian, Exponential, Arc-Cosine_1 and Arc-Cosine_2 for models M4) random forest and M5) Bayesian regression models.

## SE1. R code for model M4

## rm(list = ls())

## library(SKM)

## # Import Maize data from SKM library

## data(Maize)

## # Data preparation

## Line <- model.matrix(~0 + Line, data = Maize$Pheno)

## Env <- model.matrix(~0 + Env, data = Maize$Pheno)

## Geno <- cholesky(Maize$Geno[, -1])

## LinexGeno <- Line %*% Geno

## LinexGenoxEnv <- model.matrix(~ 0 + LinexGeno:Env)

## kernels <- c(

## "Linear",

## "Polynomial",

## "Sigmoid",

## "Gaussian",

## "Exponential",

## "Arc_cosine",

## "Arc_cosine_L"

## )

## X <- cbind(Env, LinexGeno, LinexGenoxEnv)

## y <- Maize$Pheno$Y

## for (kernel in kernels) {

## cat("*** Kernel:", kernel, " ***\n")

## arc_deep <- 1

## if (kernel == "Arc_cosine_L") {

## arc_deep <- 2

## ckernel <- "Arc_cosine"

## } else {

## ckernel <- kernel

## }

## # Compute the kernel

## X <- kernelize(X, kernel = ckernel, arc_cosine_deep = arc_deep)

## folds <- cv_random(

## records_number = nrow(X),

## folds_number = 5,

## testing_proportion = 0.2

## )

## Predictions <- data.frame()

## Times <- data.frame()

## for (i in seq_along(folds)) {

## cat("\t*** Fold:", i, " ***\n")

## fold <- folds[[i]]

## X_training <- X[fold$training, ]

## X_testing <- X[fold$testing, ]

## y_training <- y[fold$training]

## y_testing <- y[fold$testing]

## model <- random_forest(

## x = X_training,

## y = y_training,

## # Specify the hyperparameters values

## trees_number = c(100, 300, 500),

## sampled_x_vars_number = c(0.3, 0.5, 0.8),

## node_size = c(5, 10),

## tune_cv_type = "random",

## tune_folds_number = 5,

## tune_testing_proportion = 0.2,

## tune_type = "grid_search",

## tune_grid_proportion = 0.8

## )

## predictions <- predict(model, X_testing)

## FoldPredictions <- data.frame(

## Fold = i,

## Line = Maize$Pheno$Line[fold$testing],

## Env = Maize$Pheno$Env[fold$testing],

## Observed = y_testing,

## Predicted = predictions$predicted

## )

## Predictions <- rbind(Predictions, FoldPredictions)

## FoldTime <- data.frame(

## Kernel = kernel,

## Fold = i,

## Minutes = as.numeric(model$execution_time, units = "mins")

## )

## Times <- rbind(Times, FoldTime)

## }

## results_dir <- file.path("results/kernel", kernel, "M4")

## summaries <- gs_summaries(

## Predictions,

## save_at = results_dir

## )

## write.csv(

## Times,

## file = file.path(results_dir, "times.csv"),

## row.names = FALSE

## )

## }

## SE2. R code for model M5

## rm(list = ls())

## library(SKM)

## # Import Maize data from SKM library

## data(Maize)

## # Data preparation

## Line <- model.matrix(~0 + Line, data = Maize$Pheno)

## Env <- model.matrix(~0 + Env, data = Maize$Pheno)

## y <- Maize$Pheno$Y

## kernels <- c(

## "Linear",

## "Polynomial",

## "Sigmoid",

## "Gaussian",

## "Exponential",

## "Arc_cosine",

## "Arc_cosine_L"

## )

## for (kernel in kernels) {

## cat("*** Kernel:", kernel, " ***\n")

## arc_deep <- 1

## if (kernel == "Arc_cosine_L") {

## arc_deep <- 2

## ckernel <- "Arc_cosine"

## } else {

## ckernel <- kernel

## }

## # Compute the kernel

## Geno <- kernelize(

## Maize$Geno[, -1],

## kernel = ckernel,

## arc_cosine_deep = arc_deep

## )

## Geno <- cholesky(Geno)

## LinexGeno <- Line %*% Geno

## LinexGenoxEnv <- model.matrix(~ 0 + LinexGeno:Env)

## X <- list(

## Env = list(x = Env, model = "FIXED"),

## LinexGeno = list(x = LinexGeno, model = "BRR"),

## LinexGenoxEnv = list(x = LinexGenoxEnv, model = "BRR")

## )

## folds <- cv_random(

## records_number = length(y),

## folds_number = 5,

## testing_proportion = 0.2

## )

## Predictions <- data.frame()

## Times <- data.frame()

## for (i in seq_along(folds)) {

## cat("*** Fold:", i, " ***\n")

## fold <- folds[[i]]

## # This function receives the whole data and the

## # testing indices separately

## model <- bayesian_model(

## x = X,

## y = y,

## testing_indices = fold$testing,

## iterations_number = 10000,

## burn_in = 5000

## )

## # Extract the predicted values of testing

## predictions <- predict(model)

## FoldPredictions <- data.frame(

## Fold = i,

## Line = Maize$Pheno$Line[fold$testing],

## Env = Maize$Pheno$Env[fold$testing],

## Observed = y[fold$testing],

## Predicted = predictions$predicted

## )

## Predictions <- rbind(Predictions, FoldPredictions)

## FoldTime <- data.frame(

## Kernel = kernel,

## Fold = i,

## Minutes = as.numeric(model$execution_time, units = "mins")

## )

## Times <- rbind(Times, FoldTime)

## }

## results_dir <- file.path("results/kernel", kernel, "M5")

## summaries <- gs_summaries(

## Predictions,

## save_at = results_dir

## )

## write.csv(

## Times,

## file = file.path(results_dir, "times.csv"),

## row.names = FALSE

## )

## }

# Supplementary Appendix SF

In this section we provide the code for implementing six compression levels (0.5, 0.4, 0.3, 0.2, 0.1 and 0) with the Arc-Cosine_1 and Gaussian sparse kernels for models M4) random forest and M5) Bayesian regression models.

## SF1. R code for model M4

rm(list = ls())

library(SKM)

# Import Maize data from SKM library

data(Maize)

# Data preparation

Line <- model.matrix(~0 + Line, data = Maize$Pheno)

Env <- model.matrix(~0 + Env, data = Maize$Pheno)

Geno <- cholesky(Maize$Geno[, -1])

LinexGeno <- Line %*% Geno

LinexGenoxEnv <- model.matrix(~ 0 + LinexGeno:Env)

kernels <- c("Sparse_Gaussian", "Sparse_Arc_cosine")

lines_proportions <- c(0.5, 0.6, 0.7, 0.8, 0.9, 1)

X <- cbind(Env, LinexGeno, LinexGenoxEnv)

y <- Maize$Pheno$Y

for (kernel in kernels) {

cat("*** Kernel:", kernel, " ***\n")

for (line_proportion in lines_proportions) {

cat("*** Line_Proportion:", line_proportion, " ***\n")

# Compute the kernel

X <- kernelize(

X,

kernel = kernel,

arc_cosine_deep = 2,

rows_proportion = line_proportion

)

folds <- cv_random(

records_number = nrow(X),

folds_number = 5,

testing_proportion = 0.2

)

Predictions <- data.frame()

Times <- data.frame()

for (i in seq_along(folds)) {

cat("\t*** Fold:", i, " ***\n")

fold <- folds[[i]]

X_training <- X[fold$training, ]

X_testing <- X[fold$testing, ]

y_training <- y[fold$training]

y_testing <- y[fold$testing]

model <- random_forest(

x = X_training,

y = y_training,

# Specify the hyperparameters values

trees_number = c(100, 300, 500),

sampled_x_vars_number = c(0.3, 0.5, 0.8),

node_size = c(5, 10),

tune_cv_type = "random",

tune_folds_number = 5,

tune_testing_proportion = 0.2,

tune_type = "grid_search",

tune_grid_proportion = 0.8

)

predictions <- predict(model, X_testing)

FoldPredictions <- data.frame(

Fold = i,

Line = Maize$Pheno$Line[fold$testing],

Env = Maize$Pheno$Env[fold$testing],

Observed = y_testing,

Predicted = predictions$predicted

)

Predictions <- rbind(Predictions, FoldPredictions)

FoldTime <- data.frame(

Kernel = kernel,

LinesProportion = line_proportion,

Fold = i,

Minutes = as.numeric(model$execution_time, units = "mins")

)

Times <- rbind(Times, FoldTime)

}

results_dir <- file.path(

"results/sparse_kernel",

kernel,

line_proportion,

"M4"

)

summaries <- gs_summaries(

Predictions,

save_at = results_dir

)

write.csv(

Times,

file = file.path(results_dir, "times.csv"),

row.names = FALSE

)

}

}

## SF2. R code for model M5

rm(list = ls())

library(SKM)

# Import Maize data from SKM library

data(Maize)

# Data preparation

Line <- model.matrix(~0 + Line, data = Maize$Pheno)

Env <- model.matrix(~0 + Env, data = Maize$Pheno)

y <- Maize$Pheno$Y

kernels <- c("Sparse_Gaussian", "Sparse_Arc_cosine")

lines_proportions <- c(0.5, 0.6, 0.7, 0.8, 0.9, 1)

for (kernel in kernels) {

cat("*** Kernel:", kernel, " ***\n")

for (line_proportion in lines_proportions) {

cat("*** Line_Proportion:", line_proportion, " ***\n")

# Compute the kernel

Geno <- kernelize(

Maize$Geno[, -1],

kernel = kernel,

arc_cosine_deep = 2,

rows_proportion = line_proportion

)

# Sin esto porque da error.

# Geno <- cholesky(Geno)

LinexGeno <- Line %*% Geno

LinexGenoxEnv <- model.matrix(~ 0 + LinexGeno:Env)

X <- list(

Env = list(x = Env, model = "FIXED"),

LinexGeno = list(x = LinexGeno, model = "BRR"),

LinexGenoxEnv = list(x = LinexGenoxEnv, model = "BRR")

)

folds <- cv_random(

records_number = length(y),

folds_number = 5,

testing_proportion = 0.2

)

Predictions <- data.frame()

Times <- data.frame()

for (i in seq_along(folds)) {

cat("*** Fold:", i, " ***\n")

fold <- folds[[i]]

# This function receives the whole data and the

# testing indices separately

model <- bayesian_model(

x = X,

y = y,

testing_indices = fold$testing,

iterations_number = 10000,

burn_in = 5000

)

# Extract the predicted values of testing

predictions <- predict(model)

FoldPredictions <- data.frame(

Fold = i,

Line = Maize$Pheno$Line[fold$testing],

Env = Maize$Pheno$Env[fold$testing],

Observed = y[fold$testing],

Predicted = predictions$predicted

)

Predictions <- rbind(Predictions, FoldPredictions)

FoldTime <- data.frame(

Kernel = kernel,

LinesProportion = line_proportion,

Fold = i,

Minutes = as.numeric(model$execution_time, units = "mins")

)

Times <- rbind(Times, FoldTime)

}

results_dir <- file.path(

"results/sparse_kernel",

kernel,

line_proportion,

"M5"

)

summaries <- gs_summaries(

Predictions,

save_at = results_dir

)

write.csv(

Times,

file = file.path(results_dir, "times.csv"),

row.names = FALSE

)

}

}
